# Decidual Cell Polyploidization Necessitates Mitochondrial Activity

**DOI:** 10.1371/journal.pone.0026774

**Published:** 2011-10-25

**Authors:** Xinghong Ma, Fei Gao, Allison Rusie, Jennifer Hemingway, Alicia B. Ostmann, Julie M. Sroga, Anil G. Jegga, Sanjoy K. Das

**Affiliations:** 1 Division of Reproductive Sciences, Department of Pediatrics, Cincinnati Children's Hospital Medical Center, University of Cincinnati College of Medicine, Cincinnati, Ohio, United States of America; 2 Perinatal Institute, Cincinnati Children's Hospital Medical Center, Cincinnati, Ohio, United States of America; 3 Biomedical Informatics, Cincinnati Children's Hospital Medical Center, University of Cincinnati College of Medicine, Cincinnati, Ohio, United States of America; Institute of Zoology, Chinese Academy of Sciences, China

## Abstract

Cellular polyploidy has been widely reported in nature, yet its developmental mechanism and function remain poorly understood. In the present study, to better define the aspects of decidual cell polyploidy, we isolated pure polyploid and non-polyploid decidual cell populations from the *in vivo* decidual bed. Three independent RNA pools prepared for each population were then subjected to the Affymetrix gene chip analysis for the whole mouse genome transcripts. Our data revealed up-regulation of 1015 genes and down-regulation of 1207 genes in the polyploid populations, as compared to the non-polyploid group. Comparative RT-PCR and *in situ* hybridization results indeed confirmed differential expressional regulation of several genes between the two populations. Based on functional enrichment analyses, up-regulated polyploidy genes appeared to implicate several functions, which primarily include cell/nuclear division, ATP binding, metabolic process, and mitochondrial activity, whereas that of down-regulated genes primarily included apoptosis and immune processes. Further analyses of genes that are related to mitochondria and bi-nucleation showed differential and regional expression within the decidual bed, consistent with the pattern of polyploidy. Consistently, studies revealed a marked induction of mitochondrial mass and ATP production in polyploid cells. The inhibition of mitochondrial activity by various pharmacological inhibitors, as well as by gene-specific targeting using siRNA-mediated technology showed a dramatic attenuation of polyploidy and bi-nucleation development during *in vitro* stromal cell decidualization, suggesting mitochondria play a major role in positive regulation of decidual cell polyploidization. Collectively, analyses of unique polyploidy markers and molecular signaling networks may be useful to further characterize functional aspects of decidual cell polyploidy at the site of implantation.

## Introduction

Polyploidy has been widely reported to occur in a large variety of plant and animal cells. The mammalian cells, including the hepatocytes, cardiac myocytes, arterial smooth muscle cells, megakaryocytes, trophoblasts, and decidual cells [1], [2], [3], [4], all develop different degrees of polyploidy during their lifespan, although our understanding of its developmental mechanism and function in different tissues remains poor. In this regard, it is important to note that several biological processes viz., different developmental aspects [5], [6], [7], [8], [9], cellular differentiation [10], [11], cell fate acquisition and maintenance [12], tissue expansion/regeneration [13], nutritional/metabolic activity [13], [14], and embryo implantation [15] have been implicated in association with polyploidy. Although it is well recognized that for a normal cell cycle cells must receive a complete copy of their genome at each division to ensure genomic stability, studies have documented that the loss of this regulation could lead to the generation of polyploidy, by which cells undergo continuous DNA synthesis (or endocycle) without cell cytokinesis [1], [2], [3], [4], [14].

In early pregnancy, development of the differentiated uterus is critical to support embryonic growth and implantation, and the whole process is coordinately controlled by ovarian estrogen and progesterone (P_4_) [16], [17]. In the receptive uterus on day 4 of pregnancy (day 1 =  vaginal plug), the onset of embryo implantation elicits the prerequisite transformation of stromal cells into decidual cells (decidualization), which is a gateway to pregnancy establishment. The pulse-labeling experiments with ^3^H-thymidine incorporation have provided evidence that decidual cells originate from proliferating stromal cells [18]. In this regard, our recent studies in mice also showed that decidual cell transformation begins with extensive stromal cell proliferation in the morning on day 5, followed by regional differentiation into specialized type of cells (decidual cells) with acquisition of polyploidy on days 6–8 of pregnancy [11], [19]. The differentiating stromal cells, at the antimesometrial pole in close proximity to the embryo implantation, initially form the primary decidual zone (PDZ) in the afternoon on day 5. PDZ is avascular and epithelioid in nature [20]. From day 5 afternoon through day 6, stromal cells next to the PDZ continue to proliferate and differentiate into polyploid decidual cells, forming the secondary decidual zone (SDZ). SDZ is fully developed by day 7 afternoon, and at this time polyploidy development gradually spreads not only at the antimesometrial pole, but also at the lateral junctional region between the mesometrial and antimesometrial decidual poles [11]. In contrast, the mesometrial decidual cells, on days 7 and 8, continuously undergo proliferation and differentiation to form a non-polyploid decidual zone prior to placentation. The distribution pattern of polyploid decidual cells at the site of embryo implantation is also similarly exhibited in the experimentally (oil)-induced decidual bed [11]. The mechanisms regulating the regional decidual cell differentiation are complex; several signaling mediators, including homeobox transcription factors, cell-cycle molecules, cytokines, growth factors, and lipid mediators, have been implicated during the progression of decidualization [4], [21], [22], [23], [24], [25].

The formation of mono- or bi-nuclear large polyploid cells during decidualization has been well recognized in rodents [11], [26], [27], [28], [29] and recently in humans (Hirota Y and Dey SK, unpublished observation). The polyploid decidual cells are considered to be terminally differentiated cells and are developed via abnormal mitosis, but not through a simple cell fusion mechanism [11], [27], [29]. We have recently studied the aspects of cell cycle regulation for decidual polyploidy [4], [11], [29]. More specifically, studies have shown that the low accumulation of stromal cyclin D3 in the morning on day 4 is remarkably up-regulated in decidualizing stromal cells following the onset of implantation [30]. This regulation is strongly associated with stromal cell proliferation via cyclin D3/cdk4 kinase activity, while the differentiation, including terminal differentiation for polyploidy development, primarily involves a ternary complex cyclin D3/cdk6/p21 [11], [19]. The repression of a distinct set of cell cycle regulators for the G2/M phase transition appears to be associated with the onset of decidual cell polyploidy [4], [11]. Consistently, lack of cyclin D3 in mice significantly affects pregnancy outcome, with defects due to compromised decidualization and embryonic resorption [4]. Furthermore, studies have shown that HB-EGF, the earliest molecular marker of implantation [31], specifically controls stromal cell polyploidy and decidualization via overexpression of cyclin D3 [29]. Several other regulators of decidualization and embryo implantation, such as Hoxa-10 (a homeobox transcription factor) [19], [30], Stathmin (a cytosolic phosphoprotein) [32], IL11/IL11ra/BIRC5 (Survivin) signaling system [33], [34], BTEB1 (a member of the Sp/Krüppel-like family of transcription factors) [35], and DEDD (death-effector domain-containing protein) [15], have been shown to utilize cyclin D3 as a downstream effector for appropriate control of uterine decidualization. The loss of DEDD in mice results in attenuation of decidual polyploidy with severely compromised decidualization and failure of pregnancy success, implicating polyploidy development is crucially involved with decidualization during the progression of early embryo implantation [15]. In addition, Hoxa10 and cyclin D3 knock-out mouse models [4], [19], which show decidualization defect, also exhibit dramatic attenuation of polyploidy development (Gao F and Das SK, unpublished observation).

To better define the developmental and functional aspects of decidual polyploidy, we analyzed global gene expression profiling of polyploid decidual cells, as compared to non-polyploid decidual cells. Our analysis revealed an alteration of a large array of genes in association with polyploidy, and further gene enrichment analyses suggested that polyploidy is primarily enriched by multiple processes, including cell/nuclear division cycle, ATP binding, the metabolic system, and mitochondrial function, while that of depressed processes primarily include apoptosis and the immune system. Several identified genes related with mitochondria and bi-nucleation appeared to show marked increases in expression during decidualization in a regional fashion, which is consistent with the status of polyploidy. Further studies revealed that polyploid decidual cells are enriched with mitochondrial activity, and pharmacological and genetic inhibition of mitochondrial activity resulted in suppression of polyploidy development during stromal cell decidualization, suggesting mitochondria play a crucial role for polyploidization.

## Materials and Methods

### Animals and artificial decidualization

CD-1 (Charles River Laboratories, Wilmington, MA) mice were housed in the animal care facility at Cincinnati Children's Hospital Medical Center according to National Institutes of Health (NIH) and institutional guidelines for the use of laboratory animals. All protocols for the present study were reviewed and approved by the Institutional Animal Care and Use committee (IACUC) (Approval number: 1D05042). Adult females were mated with vasectomized males of the same strain to induce pseudopregnancy (day 1 =  vaginal plug). To stimulate experimentally induced decidualization (deciduoma), sesame oil (25 µl) was infused intraluminally in one uterine horn on day 4 of pseudopregnancy. Deciduoma was collected on day 7.

### Isolation of polyploid or non-polyploid decidual cells *in vivo*

Deciduomal tissues were separated from the muscle and minced by scalpel blade, and then placed in HBSS containing 0.25% collagenase, 0.1% DNase I, and 0.1% hyaluronidase for 45 min. at 37°C. The enzyme digested tissues were then serially passed through 18, 22, and 25 gauge needles to make a single cell suspension. As described previously [36], a single cell suspension in 40 ml HBSS/0.5% bovine serum albumin (BSA) (2–5×10^6^ cells/ml) was first loaded from the bottom of the sedimentation chamber (Fisher) and then loaded with BSA solution in a density gradient (2–4%) from the bottom of the sedimentation chamber at a rate of 10 ml/min. The whole operation was carried out at 4^0^ C. Cells were allowed to settle down in the gradient for a period of 3 h and then collection of the fractions began. Approximately one hundred fractions (10 ml each) were collected. Cells were isolated by centrifugation and re-suspended in freezing medium for short-term storage at −80^0^ C. The viability of the above cell preparations was close to 99%. Every fifth fractions were analyzed by phase-contrast and fluorescence (DAPI) microscopy for the presence of polyploid or non-polyploid cell populations. In general, approx. 10–15 fractions, either for polyploid or non-polyploid populations, were pooled for subsequent RNA analyses.

### Microarray and data analysis

Microarray hybridization and analysis were conducted according to the Affymetrix recommended protocols with the help of Microarray Core Facility at Cincinnati Children's Hospital Medical Center. In brief, RNAs were extracted from polyploid and non-polyploid decidual cell populations using RNeasy Mini Kit according to the manufacturer's instructions (QIAGEN, Valencia, CA). The quality of the total RNA was checked by the Agilent Bioanalyzer 2100 (Hewlett Packard) using the RNA 6000 Nano Assay. Biotin-labeled cRNAs were generated using the Affymetrix Whole Transcript Sense Target Labeling Assay (Affymetrix) and hybridized to GeneChip® mouse gene 1.0 ST array (Affymetrix Inc.). Affymetrix GeneChip Scanner 3000 7G used to scan and quantitate the gene chips using default scan settings. The raw data has been deposited to the GEO database (Accession number  =  GSE28917). Differentially expressed genes were selected based on the GeneSpring GX10 program, with a threshold of unpaired t-test (P-value ≤0.05), false discovery rate (FDR) ≤5%, and a fold change cut off  = 2.0.

Functional enrichment analysis of the differentially expressed genes from microarray data analysis was performed using the ToppFun server (http://toppgene.cchmc.org) [37] and DAVID Bioinformatics Resources 6.7 (http://david.abcc.ncifcrf.gov/) [38]. Biological association networks were built using the Ingenuity Pathway Analysis (IPA; http://www.ingenuity.com/) (Ingenuity Systems, Inc., Redwood City, CA) [39].

### Comparative RT-PCR confirmation of microarray data

RNA (1 µg total RNA from each sample) was primed with random-hexamers in a volume of 20 µl and reverse transcribed into cDNA with cDNA synthesis kits (Promega). The resulting cDNA was subjected for comparative RT-PCR analysis as previously described [19]. In brief, the comparative cDNAs were subjected to appropriate number of PCR cycle (as indicated in figures for genes of interests) for linear amplification. Initial PCR studies have determined the linear range of amplification. Amplified fragments were separated by electrophoresis on 2% agarose gels and visualized by ethidium bromide staining. The intensity of each band was measured by Scion Image (Scion Corp., Frederick, MD). The relative levels of expression of each mRNA were normalized to the expression of housekeeping gene *Actb* mRNA.

### Hybridization Probes

Mouse-specific cDNA clones (in pCRIITOPO vector) were generated by RT-PCR. The authenticity for each of these clones was confirmed by nucleotide sequencing. For *in situ* hybridization, sense and antisense ^35^S-labeled cRNA probes were generated.

### *In situ* hybridization

Frozen sections (10 µm) were hybridized with ^35^S-labeled cRNA probes as described previously [31]. Sections hybridized with sense probes served as negative controls and showed no positive signals.

### Immunohistochemistry

Immunostaining of Tdo2 and Nsbp1 was performed on formalin-fixed, paraffin-embedded tissue sections as previously described [11], [29], using specific primary antibodies to Tdo2 [40] and Nsbp1 [41].

### *In vitro* stromal cell decidualization

This procedure was followed as we previously described [29]. In brief, mouse uterine stromal cells collected on day 4 of pseudopregnancy [29] were cultured on the cover slips in presence of the growth medium [DMEM/F-12 (1∶1), plus 10% charcoal stripped serum and antibiotic]. Following a brief attachment for 1 h, cells were washed thoroughly in HBSS and replenished with the growth medium for 24 h. Cells were then replaced with the decidualization medium [1 µM P4, 10 nM E2 and 0.5% HB-EGF in DMEM/F12, plus 1% charcoal stripped FBS] for an additional 5-day period in culture. Cells were washed and replenished every alternate day. Cells were trypsinized for subsequent analysis.

### Treatment of pharmacological inhibitors for mitochondrial function during in vitro decidualization

In the above *in vitro* stromal cell decidualization, based on initial studies of cell viability with the use of various concentrations of mitochondrial inhibitors, cells were treated with vehicle or a selected concentration of different inhibitors [rotenone (0.2 µM), TTFA (80 nM), antimycin (2 µM), KCN (500 µM), CCCP (10 µM) or oligomycin (12.5 nM)] at the time of decidual stimulation for 8–12 h, and then washed the cells and kept in decidualization medium. Two-days later, cells were washed, treated with inhibitors and placed in decidualization medium as above and then cultured for three more days.

### siRNA-driven perturbation of mitochondrial genes during in vitro decidualization

In the above *in vitro* decidualization model, stromal cells, prior to the initiation of decidualization, were subjected to transfection with different siRNAs (at 100 nM) using the lipofectamine 2000 reagent (Invitrogen) for 6h, according to the manufacturer's instructions. We used two independent siRNAs for a gene specific to the mitochondrial respiratory chain complex I or complex III: *NADH dehydrogenase (ubiquinone) 1 alpha subcomplex, 4* (*Ndufa4*) or *cytochrome c-1* (*Cyc1*), respectively. Mouse specific siRNAs for *Ndufa4*: AACTATGAAGTTCACTGTAAA (siRNA1) and AAGGCCCAGACTTCTAAACTA (siRNA2), or for Cyc1: CAGCATGGATTATGTGGCGTA (siRNA1) and CAGGATGGCCCTAATGATGAT (siRNA2) were purchased from Qiagen. In parallel studies, cells were also transfected with control siRNAs (AllStars Negative Control siRNA, Qiagen).

### Mitochondrial staining

*In vivo* isolated polyploid or non-polyploid decidual cells were attached on cover slips after a brief culture for 2 h in presence of DMEM/F-12 (1∶1), plus 10% charcoal stripped serum and antibiotic. Cells were washed in HBSS (without Ca^++^ and Mg^++^) and then subjected to mitochondrial staining with 200 nM Mitotracker *Red CMXRos (Invitrogen)* for 30 min at 37°C. *In vitro* decidualized cells on the cover slips, as described above, were washed in HBSS and followed the mitochondrial staining as above. After staining, cells were washed three times with HBSS and fixed with 3.7% paraformaldehyde in HBSS for 15 min at 37°C. After fixation, cells were washed three times with HBSS and the nuclei were counter-stained with DAPI for 5 min in HBSS at 37°C. Cells were then permeabilized with ice-cold acetone for 10 minutes and rinsed once in HBSS. Cells were visualized with an inverted microscope (NIKON TE 2000U) with opti-grid structured light confocal system (Phylum) that has an all-motorized Z-focus device to capture high quality confocal images.

### Analysis of ATP content

Cellular ATP was assessed using StayBrite™ Highly Stable ATP Assay Kit (BioVision, cat# K791-100) according to the commercially available instructions.

### Quantitation of bi-nuclear cells

Cells were washed with HBSS and cyto-spun onto slides for histological analysis, as described before [29]. Approximately 500–600 cells were examined under the microscope and viewed on the computer screen. Randomly selected images from at least 20–30 different areas on the slide were captured in the computer, and bi-nuclear cell numbers were counted manually.

### Flow cytometric analysis of DNA content

Cells were fixed in 70% ethanol and then treated with RNase A (500 µg/mL) for 30 min at 37^o^C. Cells were then stained with propidium iodide (PI, 50 µg/mL) and directly analyzed for the DNA content by flow cytometry (BD FACSCanto II) using the Cincinnati Children's Core facility. At least 50,000 to 100,000 cells were subjected for each analysis.

## Results

### Bovine serum albumin (BSA) density gradient cell sedimentation technique allows successful isolation of polyploid and non-polyploid decidual cells

**Figure 1:**
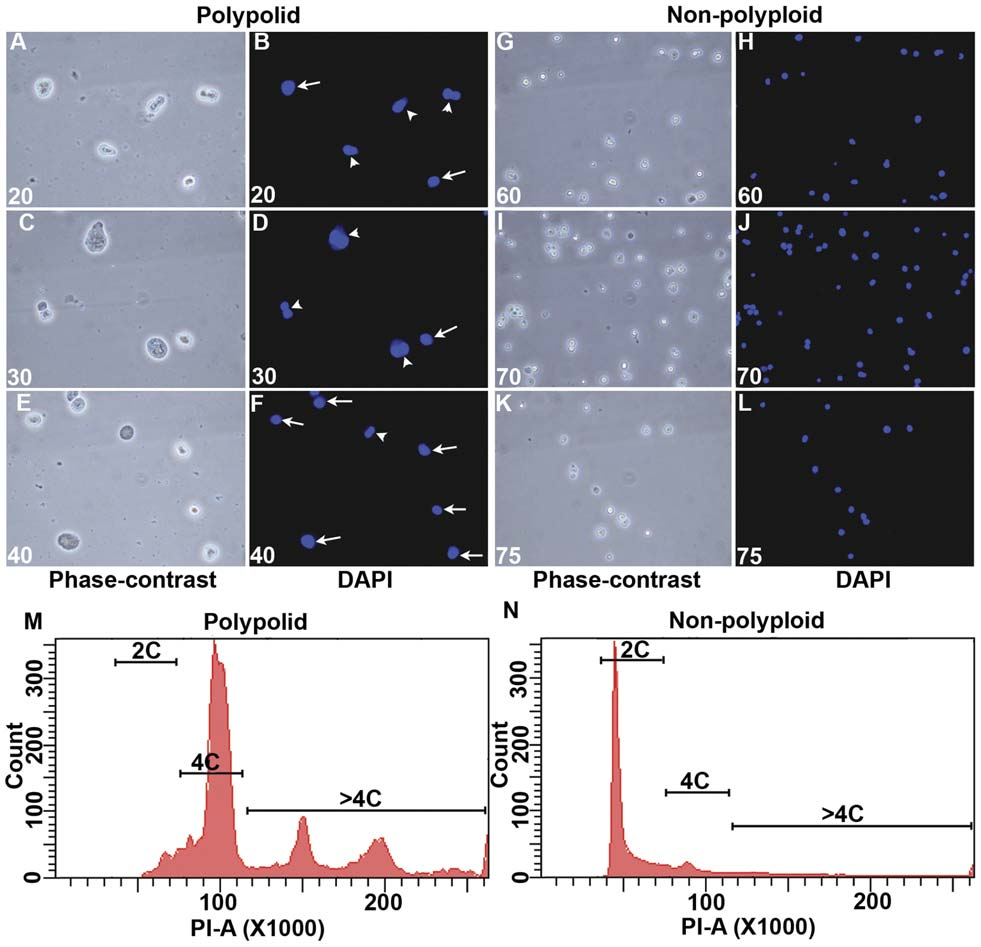
Analysis of polyploid and non-polyploid cells. Deciduomal cells collected on day 7 of pseudopregnancy were fractionated by BSA density gradient (2–4%), as described in Materials and Methods. Representative phase-contrast and fluorescence (DAPI) microscopic pictures are shown in parallel for polyploid (A–F) and non-polyploid (G–L) populations collected in fraction numbers 20–40 and 60–75, respectively. Pictures are at 400X. Note: Polyploid cells are identified by the presence of large mono-nuclear (shown by arrows) or bi-nuclear (shown by arrowheads) structures, while the non-polyploid cells are devoid of such nuclear structures. Flow cytometric analyses of the DNA content for a representative polyploid (M) and non-polyploid (N) population are shown. Note: The polyploid fractions are enriched with DNA content ≥4N, while the non-polyploid fractions are primarily devoid of cells with DNA content >4N. These experiments were repeated at least three times with similar results.

In order to examine the status of the gene expression profile during the progression of decidual cell polyploidy, we first focused on the isolation of enriched preparations of polyploid and non-polyploid decidual cells, collected during an optimal time of polyploidization on day 7 of pseudopregnancy, following artificial stimulation for uterine decidualization in mice [11]. Deciduomal tissues were digested with enzymes to obtain a single cell suspension and then subjected to the cell separation technique, as described in Materials and Methods. Based on initial phase-contrast and fluorescence (DAPI) microscopic analyses, fractions collected between 20–40 were found to contain polyploid cells, albeit at the level of 97% purity, as judged by the presence of large mono- or bi-nuclear cells (Fig. 1, A–F), while fractions between 60–75 were found to be enriched with non-polyploid cells, at the level of 98% purity (Fig. 1, G–L). Further analysis of the expression of decidualizing stromal cell marker with cyclin D3 appears to indicate that the cell preparations were indeed very pure (Fig. S1). In addition, flow cytometric analysis of DNA content also clearly revealed that polyploid fractions are primarily enriched with DNA content ≥4N (Fig. 1M), while non-polyploid fractions are primarily devoid of cells containing DNA content >4N (Fig. 1N). Thus, our subsequent studies utilized the pooled fractions collected between 20–40 or 60–75 to represent polyploid or non-polyploid cell populations, respectively.

### Microarray analysis identifies differentially modulated genes in polyploid decidual cells as compared to non-polyploid decidual cells

We next examined the differential gene expression profile in the above polyploid (P) and non-polyploid (N) cell populations to gain insights into the signaling networks that might be important for the developmental regulation of polyploidy during decidualization. We collected three independent pools of P- and N-samples, and total RNAs were isolated and subjected to gene expression profiling utilizing the GeneChip® mouse gene 1.0 ST array (Affymetrix Inc.), which offers whole genome transcript coverage. Our analysis revealed that a total 2222 genes (P value ≤0.05) were differently expressed in P as compared to N (Table S1). Of the total 2222 genes, 1015 genes exhibited an increase in expression for polyploid cells, whereas 1207 genes were depressed in polyploid cells, suggesting that a drastic alteration of gene expression occurs during the transition from non-polyploid to polyploid cell populations. Furthermore, the cluster analysis of the microarray data for differential gene expression indicated that they were indeed segregated into two categories, as judged by distinct molecular signatures (Fig. S2). Overall, these results suggest that polyploid decidual cell populations are markedly different in respect to gene expression profiles when compared to non-polyploid decidual cell populations.

### RT-PCR analysis validates microarray data

**Figure 2:**
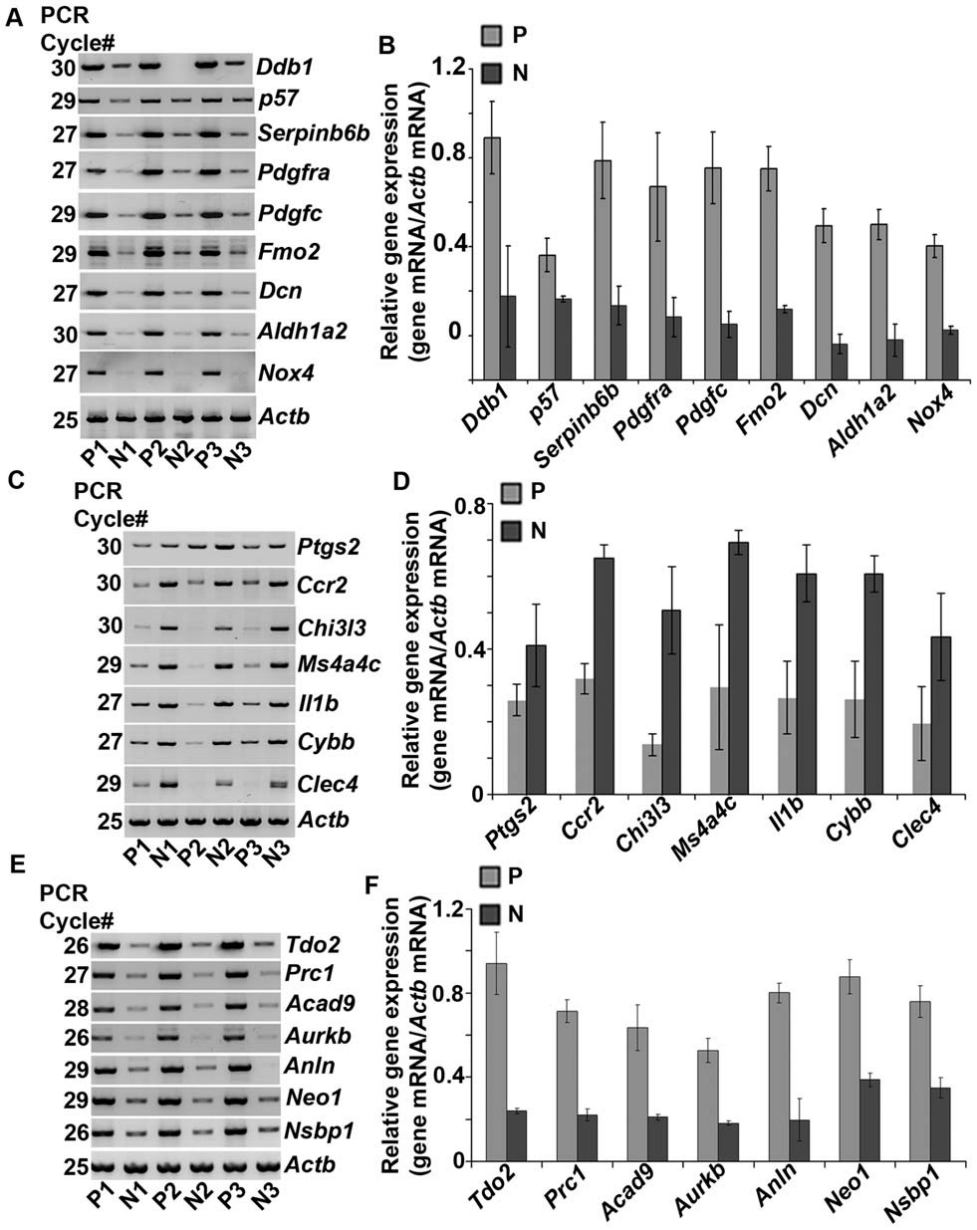
Analysis of expression for differentially modulated polyploidy-related genes. Comparative RT-PCR: Upregulation (A), downregulation (C) and bi-nuclear (E) genes. Total RNA was extracted from three independent pools of polyploid (P1, P2 and P3) and non-polyploid (N1, N2 and N3) populations, and then subjected to RT-PCR at indicated PCR cycle numbers to achieve linear amplification for genes of interest. Amplified DNA bands were visualized by ethidium bromide staining. Quantitative analysis: Relative levels of expression for upregulation (B), downregulation (D) and bi-nuclear (F) genes are shown. Band intensities shown in (A), (C), and (E) were measured by densitometric analysis and relative levels of gene-specific mRNAs were obtained after correction with *Actb*. These experiments were repeated at least three times with similar results.

In order to validate the above microarray results, we analyzed the expression between the three independent P- and N-samples for randomly selected up-regulated (Fig. 2A, B) and down-regulated genes (Fig. 2C, D) by comparative RT-PCR analysis, as described in the Materials and Methods. The primers used for RT-PCR and the sizes of PCR products are indicated in Table S2. The RT-PCR derived products were confirmed by cloning and sequencing. Band intensities were measured by densitometric analysis and corrected against *Actb* (Fig. 2B, D). Consistent with our microarray data, the expression of *Ddb1, P57, Serpinb6b, Pdgfra, Pdgfc, Fmo2, Dcn, Aldh1a2,* and *Nox4* was up-regulated, whereas that of *Ptgs2*, *Ccr2, Chi3l3, Ms4a4c, Il1b, Cybb,* and *Clec4c* was down-regulated in P, as compared to N. These results suggest that our experimental strategy was able to selectively identify differentially expressed genes during the onset of decidual cell polyploidy.

Recently, an analysis of cell-division phenotype, based on RNAi technology in conjunction with time-lapse computer imaging, revealed a gene list encompassing approx. 400 genes that are specifically related to the event of cell bi-nucleation [42]. Because decidual polyploid cells are associated with bi-nucleation, a direct comparison of our microarray data and the available bi-nucleation data set has allowed us to generate a list of genes that are specifically overlapped during the development of decidual cell bi-nucleation. As shown in Table 1, our analysis indicates that 23 up-regulated and 15 down-regulated genes appeared to be represented in P, as compared to N (Table 1). Among these genes, we again randomly selected a few more genes to further validate the expression by comparative RT-PCR. Our analysis revealed that the expression of *Tdo2, Prc1, Acad9, Aurkb, Anln, Neo1,* and *Nsbp1* was indeed consistent with the upregulation in P as compared to N (Fig. 2E, F). Overall, these results suggest that our microarray-identified genes appear to represent true differential expression status with the progression of decidual cell polyploidy.

**Table 1:** Genes that are specifically modulated in bi-nucleated polyploid decidual cells.

Up-regulated genes in polyploid cells	*Dgat2, Acad9, Katnal1, Ryk, Aurkb, Gemin6, Adam19, Mastl, Idh1, Prmt1, Bub1, Ect2, Crabp1, Anln, Rcn3, Usp1, Atoh8, Prc1, Ccnb1, Tdo2, Neo1, Lbp, Nsbp1*
Down-regulated genes in polyploid cells	*Kcnk6, Glrx, Cotl1, Myd88, Ncf2, Itga6, Mcam, Bcl6, Plcb2, Fxyd5, Rasgrp3, Ptafr, Ly86, Ccrl2, Fpr1*

### Polyploidy-related genes are expressed in a regional fashion within the uterine decidual bed

**Figure 3:**
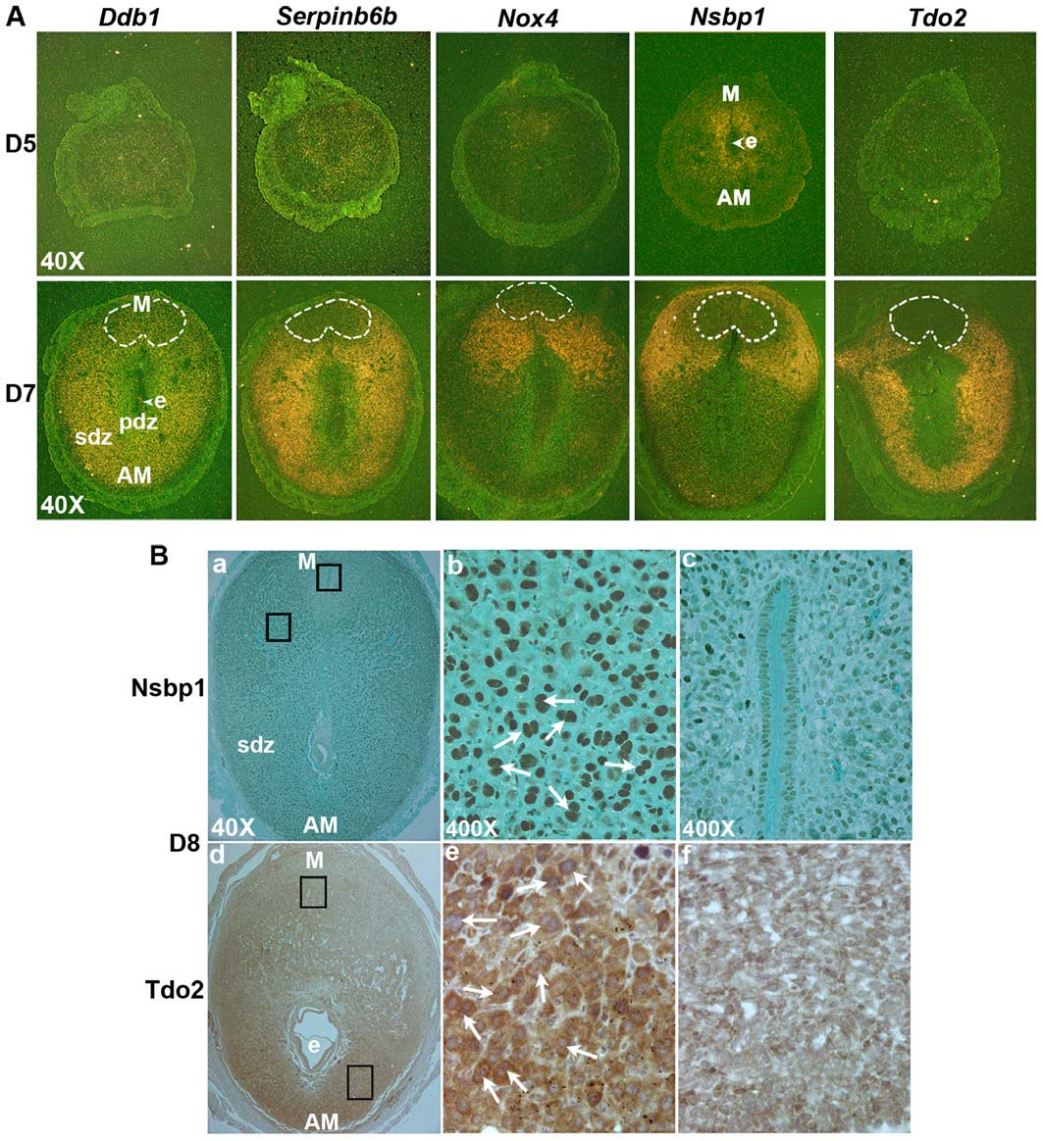
Regional distribution of expression for up-regulated polyploidy genes during decidualization at the site of implantation. (A) *In situ* hybridization: Expression of *Ddb1, Serpinb6b, Nox4, Nsbp1,* and *Tdo2* genes at the embryo implantation sites on days 5 (D5) and 7 (D7) of pregnancy is shown. Frozen sections were hybridized with ^35^S-labeled antisense or sense riboprobes, and RNase A resistant hybrids were detected by autoradiography. Sections were post-stained with hematoxylin and eosin. Dark-field photomicrographs of representative cross-sections hybridized with antisense probes are shown at 40X. Sections hybridized with corresponding sense probes did not show any positive signals (data not shown). M, mesometrial pole; AM, anti-mesometrial pole; e, embryo; pdz, primary decidual zone; sdz, secondary decidual zone. (B) Immunohistochemical analysis: Localization of Nsbp1 and Tdo2 localization at the embryo implantation sites on day 8 (D8) is shown. Arrows indicate positive nuclear or cytoplasmic staining for the localization of immunoreactive Nsbp1 or Tdo2 proteins on polyploid cells, respectively. No immunostaining was noted when similar sections were incubated with pre-immune serum (data not shown). M, mesometrial pole; AM, anti-mesometrial pole; e, embryo; sdz, secondary decidual zone. The insets shown in the mesometrial poles in panels a, d (at 40X) are presented in the respective right panels: c, f (at 400X). The other insets shown in panels: a, d, corresponding to the junctional barrier region between the mesometrial and antimesometrial poles or the region in sdz, are presented in the respective middle panels: b, e (at 400X). These experiments were repeated at least three times with similar results.

Based on the above findings, polyploidy-related upregulation (e.g., *Ddb1*, *Serpinb6b,* and *Nox4*), downregulation (*Ptgs2*), and bi-nucleation (e.g., *Nsbp1* and *Tdo2*) genes were further analyzed by *in situ* hybridization for expression during decidualization. Because, at the site of embryo implantation on the morning on day 5, decidualizing stromal cells actively participate in proliferation without showing any sign of polyploidy, and on day 7 those cells optimally undergo terminal differentiation with regional development of polyploidy [11], we analyzed expression at the sites of implantation on days 5 and 7. In general, our analysis revealed that the expression of all the above upregulation or bi-nucleation genes was minimally detected on day 5, with the exception of *Nsbp1*, which showed localized signal in the proliferating stroma primarily in close proximity around the embryo implantation (Fig. 3A). In contrast, *Ptgs2*, a down-regulated gene, was primarily detected with signals in the thin layer of stromal cells around the embryo implantation (Fig. S3). While on day 7, the expression of *Ddb1, Serpinb6b, Nox4, Nsbp1,* and *Tdo2* was primarily revealed by differential upregulation in a regional fashion within the decidual bed, with an apparent suppression in the mesometrial pole (Fig. 3A). More specifically, *Nsbp1* and *Nox4* showed predominant expression at the lateral junctional areas between the mesometrial and anti-mesometrial poles of the implantation site, although scattered low accumulation of signals also persisted in the SDZ (Fig. 3A). In addition, a distinct accumulation of signals for *Nsbp1* mRNAs was noted in a few layers of sub-myometrial decidual cells at the mesometrial pole (Fig. 3A). In contrast, *Tdo2, Serpinb6b,* and *Ddb1* showed wider distribution in the antimesometrial and mesometrial-antimesometrial junctional zone of the decidual bed (Fig. 3A). Consistent to previous results [43], the expression of *Ptgs2* was primarily exhibited in the mesometrial pole of the decidua (Fig. S3).

We next examined the localization of immunoreactive proteins for Nsbp1 and Tdo2 at the sites of embryo implantation. Consistent with the above results, distinct nuclear staining for Nsbp1 was primarily noted in polyploid decidual cells, in the mesometrial and antimesometrial junctional zones, as well as a scattered distribution in the SDZ polyploid cells, on days 7 (data not shown) and 8 (Fig. 3B) of pregnancy. The mesometrial decidual cells did not reveal any specific accumulation of this protein (Fig. 3Bc). This pattern of expression is consistent with the expression of cyclin D3, a marker of polyploidy, on day 8 (Fig. S4). In the case of Tdo2, similar to the above, cytoplasmic staining for this protein is detected primarily throughout the antimesometrial and mesometrial-antimesometrial junctional decidual bed, particularly in association with the polyploid decidual cells at the site of implantation on days 7 (data not shown) and 8 (Fig. 3C). Furthermore, both Nsbp1 and Tdo2 also revealed similar results in the experimentally induced deciduoma on days 7 and 8 of pregnancy (data not shown). Overall, these results suggest that polyploidy/bi-nucleation related genes are specifically induced in regional polyploid cells within the decidual bed.

### Multiple molecular signaling networks are differentially modulated in polyploid decidual cells

**Figure 4:**
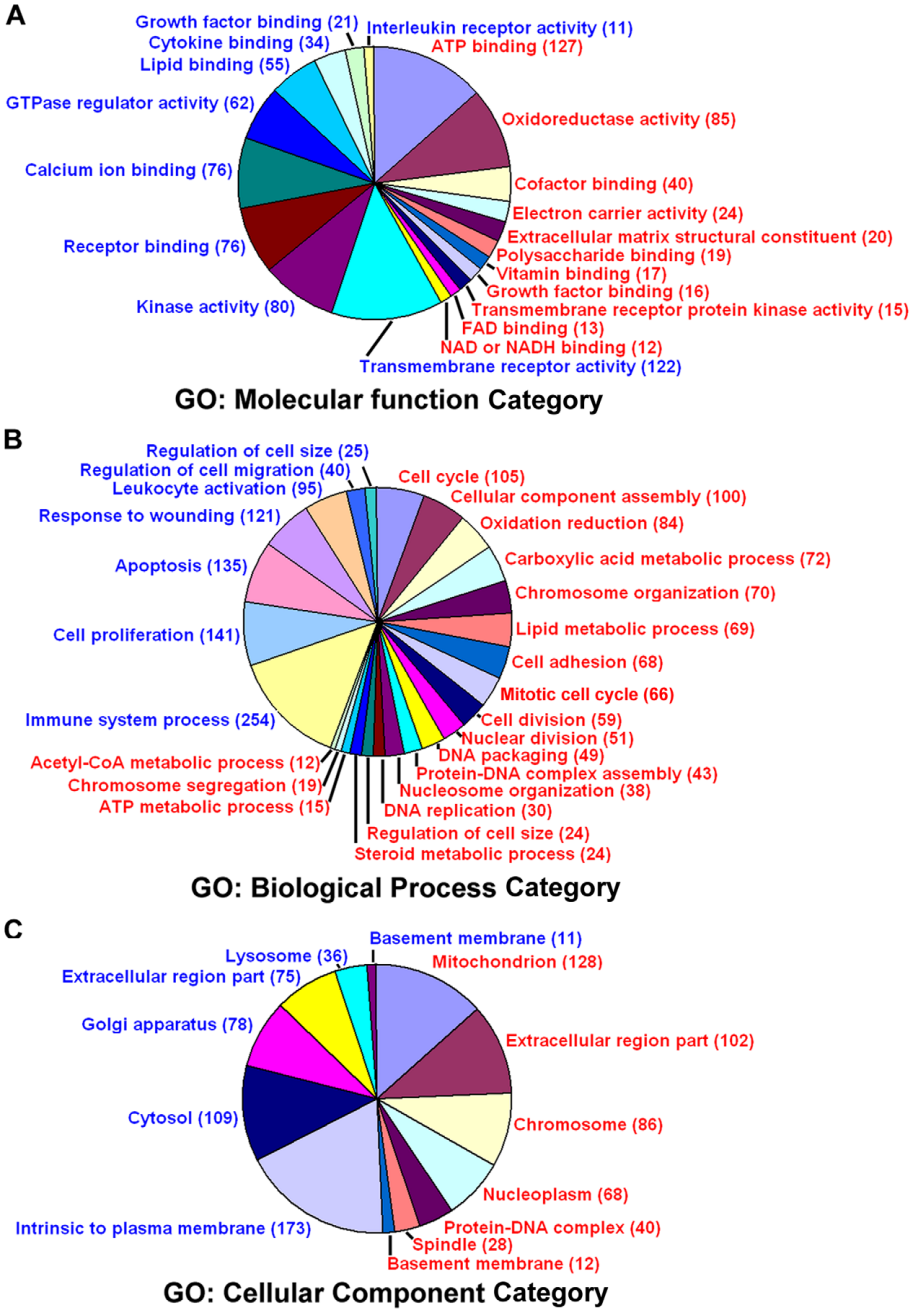
Functional categorization of differentially expressed polyploidy decidual genes. Based on ToppFun and DAVID analyses, the up- and down-regulated polyploidy genes were divided according to gene ontology (GO) terms: molecular function, biological processes, and cellular component categories. In each category, further classification of various sub-categories are represented in red, for the up-regulated genes and blue for the down-regulated genes. The numbers within the parentheses indicate the total number of genes modulated under this sub-category.

To gain further insights into the functions of differentially expressed genes, the functional enrichment analysis was carried out separately for the up- and down-regulated genes using the ToppFun and DAVID programs (Tables S3 and S4, respectively) [37], [38]. A summarized version of the predominantly altered signaling networks is represented in a graphical form (Fig. 4). Our analysis primarily categorized the genes with “Gene Ontology (GO)” terms (molecular function, biological process, and cellular component) as well as by other functional categories, such as “human phenotype,” “mouse phenotype,” “pathway,” “interaction,” “TFBS,” “coexpression,” “microRNA,” “domain,” “drug,” “gene family,” and “disease” (Tables S3 and S4). Under the GO: molecular function category, the up-regulated polyploidy genes consisted of major sub-categories that primarily include ATP binding, oxidoreductase activity, cofactor binding, electron carrier activity, extracellular matrix structural constituent, polysaccharide binding, vitamin binding, growth factor binding, transmembrane receptor protein kinase activity, FAD binding, and NAD/NADH binding, with the alteration of 127, 85, 40, 24, 20, 19, 17, 16, 15, 13, and 12 genes, respectively (Fig. 4 and Table S3). Whereas, that of down-regulated genes primarily revealed kinase activity, receptor binding, calcium ion binding, GTPase regulator activity, lipid binding, cytokine binding, growth factor binding, and interleukin receptor activity as major sub-categories with corresponding alterations of 80, 76, 76, 62, 55, 34, 21, and 11 genes (Fig. 4 and Table S4). For the GO: biological process category, up-regulated polyploidy genes primarily sub-categorized into cell cycle (105 genes), cellular component assembly (100 genes), oxidation and reduction (84 genes), carboxylic acid metabolic process (72 genes), lipid metabolic process (69 genes), cell adhesion (68 genes), steroid metabolic process (24 genes), ATP metabolic process (15 genes), and acetyl-CoA metabolic process (12 genes) (Fig. 4 and Table S3). It is interesting to note that consistent with the status of cycle regulation in polyploid decidual cells [4], the cell cycle sub-category appeared to reflect several other groups such as chromosome organization, mitotic cell cycle, cell division, nuclear division, DNA packaging, protein-DNA complex assembly, nucleosome organization, DNA replication, regulation of cell size, and chromosome segregation with corresponding alterations of 70, 66, 59, 51, 49, 43, 38, 30, 24, and 19 genes (Fig. 4 and Table S3). In contrast, the down-regulated polyploidy genes under the GO: biological process category primarily revealed that immune system process (254 genes), cell proliferation (141 genes), apoptosis (135 genes), regulation of cell migration (40 genes), and regulation of cell size (25 genes) are the major affected sub-categories (Fig. 4 and Table S4). In the case of GO: cellular component, the up-regulated polyploidy genes primarily included mitochondrion (128 genes), extracellular region part (102 genes), chromosome (86 genes), nucleoplasm (68 genes), protein-DNA complex (40 genes), spindle (28 genes), and basement membrane (12 genes) (Fig. 4 and Table S3), while that of down-regulated genes predominantly comprised intrinsic to plasma membrane (173 genes), cytosol (109 genes), Golgi apparatus (78 genes) and extracellular region part (75 genes), lysosome (36 genes), and basement membrane (11 genes) as the major sub-categories (Fig. 4 and Table S4). Furthermore, among the rest of the categories, it is interesting to note that the “mouse phenotype” revealed two major sub-categories, reproductive system phenotype (110 genes) and embryonic lethality (109 genes), while the “human phenotype” category primarily implicated “metabolism abnormality” (103 genes) for the up-regulated polyploidy genes (Table S3). Additionally, the “pathway” category for up-regulated polyploidy genes primarily represented aurora B kinase signaling (17 genes), valine/leucine/isoleucine degradation (15 genes), FOXM1 transcription factor network (14 genes), pyruvate metabolism (13 genes), tryptophan metabolism (12 genes), and citrate cycle (TCA cycle) (12 genes) (Table S3). In contrast, the down-regulated polyploidy genes under the “pathway” category primarily revealed cytokine-cytokine receptor interaction (49 genes), MAPK signaling pathway (39 genes), and natural killer cell mediated cytotoxicity (33 genes) (Table S4). Given an essential role for progesterone during decidualization, the “drug” category identified “progesterone” as a major sub-category for both up- and down-regulated polyploidy genes, with corresponding alterations of 165 and 114 genes (Tables S3 and S4, respectively). Additionally, growth regulatory molecules such as: estrogens (genistein, estradiol, bisphenol A, diethylstilbestrol, etc.), cancer growth regulating agents (doxorubicin, raloxifene, etc.), oxidants/antioxidants (hydrogen peroxide, resveratrol, paraquat, etc.), or anti-inflammatory agents (indomethacine, corticosterone, etc.) are all highlighted under the “drug category” for both up- or down-regulated polyploidy genes (Tables S3 and S4, respectively). Overall, the above analyses suggest that multiple molecular signaling networks (cell/nuclear division cycle, metabolic process, mitochondrial activity, and ATP binding for the up-regulated genes, and apoptosis and immune processes for the down-regulated genes) are affected during the transitional development of decidual cell polyploidy.

### Polyploid decidual cells exhibit enhanced mitochondrial activity

Because the above studies suggest that mitochondria might be important for the developmental control of polyploidy, we next wanted to define the mitochondrial gene networks and their functional relationships based on the above differentially altered 128 mitochondrial genes by utilizing the Ingenuity Pathway Analysis (IPA) [39]. As shown in Fig. S5, our analysis revealed that seven individual networks and an overlapping network were primarily represented by those mitochondrial genes. The particular genes involved in each network are also listed in Table 2. Overall, these networks primarily suggest that lipid metabolism, carbohydrate metabolism, cellular assembly and organization, genetic disorder, metabolic disease, small molecule biochemistry, and drug metabolism are potential mechanisms in relation to polyploidy development.

**Table 2:** Mitochondrial gene networks generated by IPA for genes that are specifically enriched in polyploidy cells.

ID	Molecules in Network	Score	Focus Molecules	Top Functions
1	*ACAT2, AIFM1, aldehyde dehydrogenase (NAD), ALDH, ALDH3A2, ALDH6A1, ALDH7A1, ALDH9A1, Ant, Beta Tubulin, Caspase, CCT7, CYC1, CYCS (includes EG:54205), CYP11A1, Cytochrome c, DLAT, FDX1, FSH, GOT2, HK2, Hsp70, HSPA9, Lh, NFkB (complex), PDHB, PEBP1, PPIF, PRDX2, PRDX3, SLC25A4, SLC25A13, TFRC, TOMM40, VDAC1*	53	25	Lipid Metabolism, Small Molecule Biochemistry, Cellular Assembly and Organization
2	*ACACA, ACAT1, adenosine-tetraphosphatase, ATP5B, ATP5D, ATP5G3, C1QBP, FASN, GJA1, H+-transporting two-sector ATPase, hCG, IDH1, IDH2, IDH3A, IDH3G, Insulin, Isocitrate dehydrogenase, MAOB, ME1, NADH dehydrogenase, NADH2 dehydrogenase, NADP Isocitrate Dehydrogenase, NDUFA4, NDUFA10 (includes EG:4705), NDUFAB1, NDUFB6, NDUFB9, NDUFS1, NDUFV1, Pkc(s), PP1 protein complex group, PP2A, PRKCA, SLC6A2, T3-TR-RXR*	47	23	Carbohydrate Metabolism, Small Molecule Biochemistry, Lipid Metabolism
3	*ACO2, ACSS3, AHSA1, GSTZ1 (includes EG:2954), HNF4A, KARS, MCCC1, MRPL18, MRPL51, MT-ND2, MT-ND3, MT-ND6, MT-ND4L, MTHFD1, NADH2 dehydrogenase (ubiquinone), NDUFA4, NDUFA7, NDUFA10 (includes EG:4705), NDUFAB1, NDUFB6, NDUFS1, NDUFS2, NDUFS3, NDUFS4, NDUFS5, NDUFS8, NDUFS6 (includes EG:4726), NDUFV1, PABPC4, PHB2, RPS6KA6, SQRDL (includes EG:58472), SUCLA2, USMG5, USP15*	39	20	Genetic Disorder, Metabolic Disease, Cardiovascular Disease
4	*ABAT, ACAT2, ACSL4, AMD1, APP, ATAD3A, AVPR1A, CRK, DBN1, EIF4ENIF1, FBLN5, FDPS, Fe2+, GANAB, GART, GLUL, HMG CoA synthase, HMGCS2, hydrogen peroxide, IDH2, LONP1, MMP10, MT-COI, MYC, NARS, PDGF BB, PITRM1, PRDX2, pregnenolone, PTRF, RSAD2, SARDH, SFXN1, TTF1, ZNRF1*	30	17	Drug Metabolism, Molecular Transport, Lipid Metabolism
5	*ACOT2, AMH, ATIC, beta-estradiol, CAPRIN2, CEBPB, Cg, CLPP, CYP11A1, CYP11B1, EGF, ETFB, FBLN1, FGF9, GARS, HSD11B1, IDH2, IDI1, IGFBP4, L-triiodothyronine, LHCGR, LIMK2, ME1, NR0B1, NR5A2, OXCT1, PCNA, PDK4, POLD2, POLD4, POLDIP2, pregnenolone, progesterone, SELENBP1, TUFM*	26	15	Small Molecule Biochemistry, Lipid Metabolism, Drug Metabolism
6	*8-oxo-7-hydrodeoxyguanosine, ABCB6, ABCC9, AK1, BID, CAT, CLYBL, COQ7, CPT2, CTSL1, CTSS, CYP11A1, ELN, EMILIN1, FBLN2, FBLN5, FCN1, Gelatinase, GLB1, HIBADH, HK2, HNF1A, HSPG2 (includes EG:3339), KCNJ11, KLF11, LDHB, LOXL1, MMP10, OGDHL, SERPINF1, SLC27A1, SLIT3, SUOX, TGFB1, TNF*	21	14	Tissue Development, Cardiovascular System Development and Function, Organismal Development
7	*ACAA2, acad, ACAD8, ACAD9, ACADM, ACADSB, acyl-CoA dehydrogenase, AIFM3, ALDH9A1, AP1M2, CTDSP1, CYBA, DHCR24, F7, GCDH, GPT2, HADH, IDH3B, IGFBP7, LMNB1, ME2, MIR124, MIR124-1 (includes EG:406907), NDOR1, oxidoreductase, OXNAD1, POR, RDH13, SLC2A4, STOM, SUCLG2, TEAD1, TSC22D4*	18	11	Genetic Disorder, Metabolic Disease, Small Molecule Biochemistry

**Figure 5:**
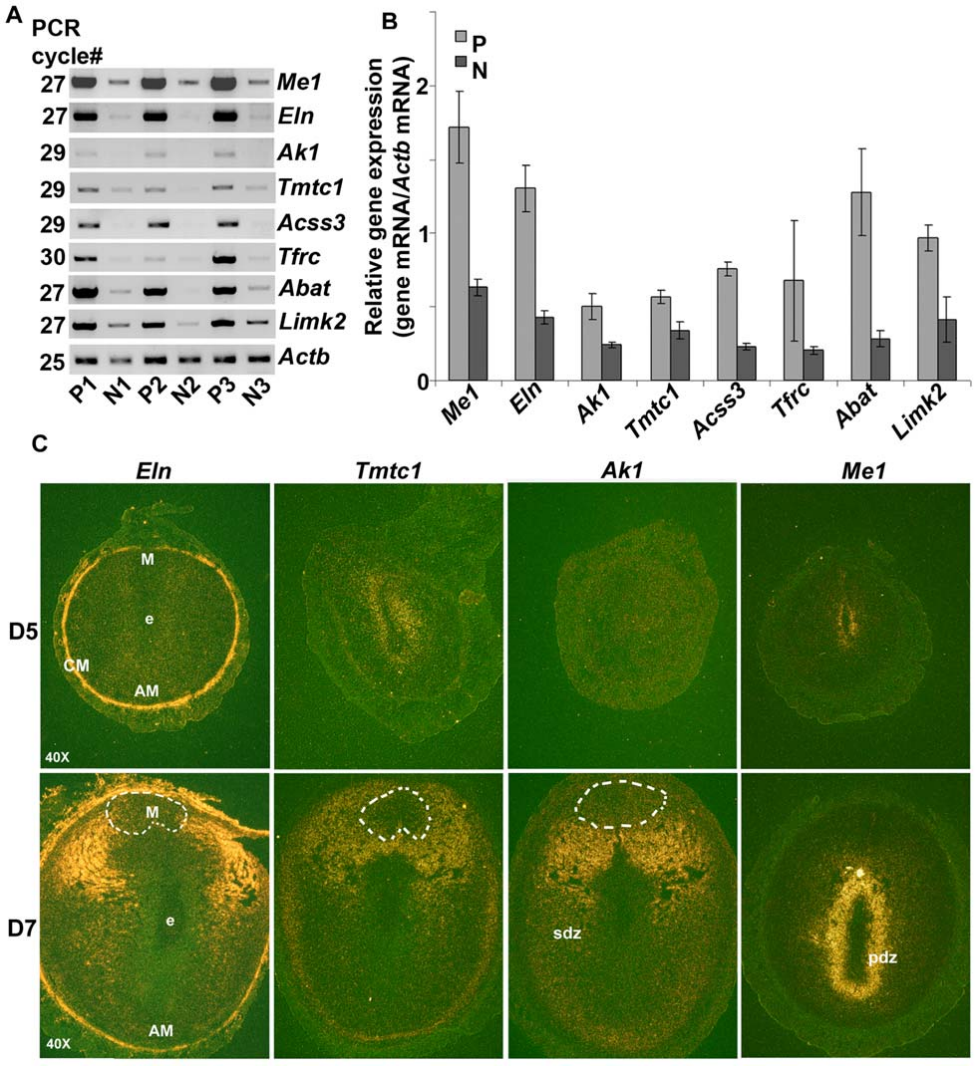
Analysis of expression for mitochondrial genes. (A) Comparative RT-PCR: Total RNA extracted from three independent samples of polyploid (P1, P2 and P3) and non-polyploid (N1, N2 and N3) populations was subjected to RT-PCR at indicated PCR cycle numbers to achieve linear amplification for genes of interest. Amplified DNA bands were visualized by ethidium bromide staining. (B) Quantitative analyses: Relative levels of gene expression for the corresponding changes in polyploid and non-polyploid samples are shown. The band intensities were measured by densitometric analyses, and relative levels of mRNAs for gene-specific expression were determined after correction with *Actb*. These experiments were repeated at least three times with similar results. (C) *In situ* hybridization: Expression of *Eln, Tmtc1, Ak1,* and *Me1* genes at the sites of embryo implantation on days 5 (D5) and 7 (D7) of pregnancy is shown. Frozen sections were hybridized and developed as described in Fig. 3A. Dark-field photomicrographs of representative uterine cross-sections hybridized with antisense probes are shown at 40X. Sections hybridized with corresponding sense probes did not show any positive signals (data not shown). M, mesometrial pole; AM, anti-mesometrial pole; e, embryo; pdz, primary decidual zone; sdz, secondary decidual zone; CM, circular muscle. These experiments were repeated at least three times with similar results.

We next validated the differential status of expression for the above mitochondrial genes by randomly selecting at least one from each of the different networks in Table 2. Consistent with our microarray results, the comparative RT-PCR analysis showed that the expression of *Me1, Eln, Ak1, Tmtc1, Acss3, Tfrc, Abat,* and *Limk2* mRNAs was indeed up-regulated in polyploid (P) samples as compared to non-polyploid (N) samples (Fig. 5A, B). Further analysis of cell-specific expression of *Eln, Tmtc1, Ak1,* and *Me1* by *in situ* hybridization at the sites of implantation on days 5 and 7 (Fig. 5C) revealed that low accumulation of signals for *Tmtc1* and *Me1* mRNAs were noted around the site of implantation in the sub-luminal stroma on day 5. However, on this day, *Eln* and *Ak1* did not exhibit any signals in the decidualizing stroma, although marked accumulation of *Eln* mRNAs was detected in the circular muscle (Fig. 5C). In contrast, on day 7, with the development of polyploidy at the site of implantation, the signals for *Eln, Tmtc1,* and *Ak1* mRNAs were exhibited in a region-specific manner within the decidual bed (Fig. 5C). More specifically, distinct signals were exhibited primarily in the barrier region between the mesometrial and antimesometrial poles, although low accumulation was also noted in the SDZ (Fig. 5C). Additionally, signals in the circular muscle or in the sub-myometrial non-differentiated stroma for *Eln* or *Tmtc1*, respectively, were also revealed on day 7 (Fig. 5C). In the case of *Me1*, robust accumulation of signals was primarily noted in the barrier region between the SDZ and PDZ (Fig. 5C). Overall, these results suggest that mitochondrial genes are differentially expressed in a regional manner within the decidual bed.

**Figure 6:**
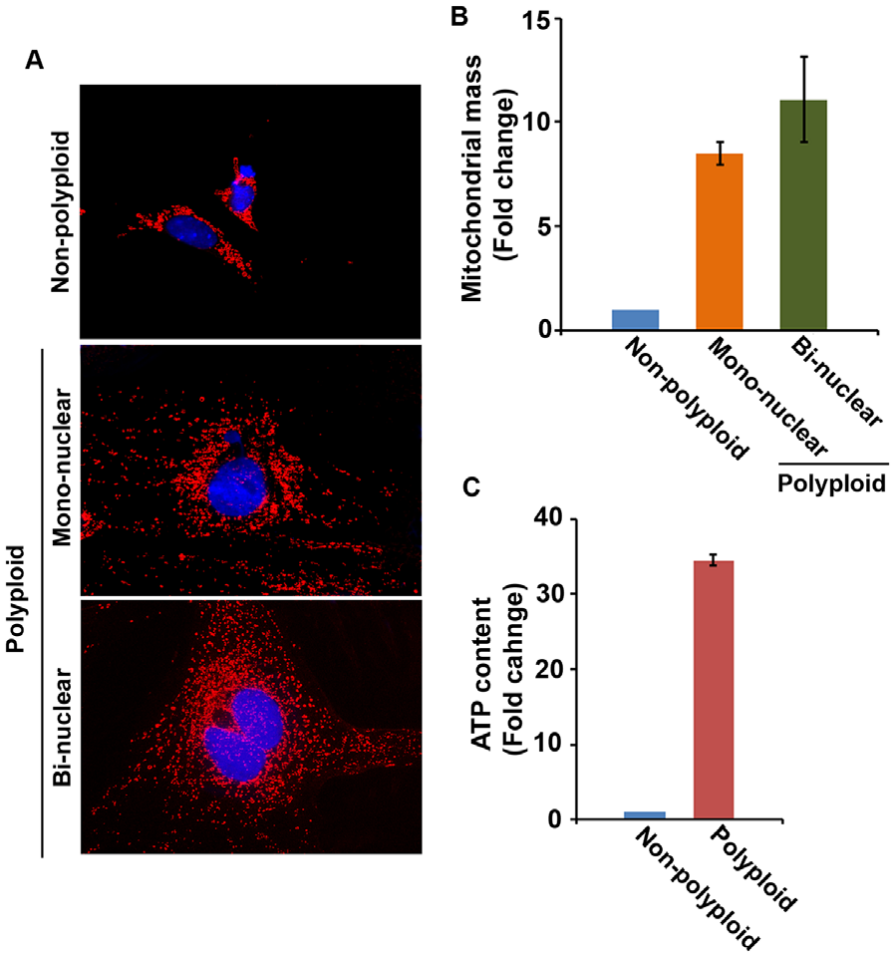
Analysis of mitochondria in relation to decidual cell polyploidy. (A) Mitochondrial mass analysis by confocal microscopy. Pure polyploid and non-polyploid decidual cells isolated from day 7 deciduomal tissues were analyzed by staining with Mitotracker *Red*, as described in Materials and Methods. (B) Quantitation of mitochondrial mass: The area of mitochondrial staining per cell was determined using the Image J program available at http://imagej.nih.gov/ij (NIH, USA). Results are expressed as fold change (mean ± SEM), as compared to non-polyploid cells (control). Data were analyzed after counting of at least 30 to 40 cells in each group from three independent experiments. (C) Analysis of ATP content: Pure polyploid and non-polyploid decidual cells were used to determine the endogenous levels of ATP, as described in the Materials and Methods. Results are expressed as fold change (mean ± SEM), as compared to non-polyploid cells (control) from three independent experiments.

We next wanted to examine the status of mitochondria in the polyploidy and non-polyploid samples, following staining with the mitochondria-specific fluorescent mitotracker Red CMXRos. Confocal microscopic analyses of the purified *in vivo* developed polyploid and non-polyploid cells revealed a dramatic upregulation of mitochondrial mass in both the bi-nuclear (11 fold) and mono-nuclear (8.5 fold) polyploid cells, as compared to non-polyploid cells (Fig. 6A, B). Our analyses of polyploidy for the mono-nuclear and bi-nuclear cells revealed a distribution of approximately 20% and 80%, respectively. Further analysis of ATP content in the above polyploidy and non-polyploid cells also revealed similar results with upregulation of ATP in polyploid cell populations (34 fold), as compared to non-polyploid cells (Fig. 6C). Furthermore, it is interesting to note that our analysis of mitochondria for *in vitro* developed decidual polyploid (bi-nuclear or mono-nuclear) cells also revealed above similar results, as compared to non-polyploid cells (Fig. S6). Overall, these results suggest that the mitochondrial activity plays a role in the development of decidual cell polyploidy.

### Mitochondrial perturbation causes attenuation of decidual cell polyploidy

**Figure 7:**
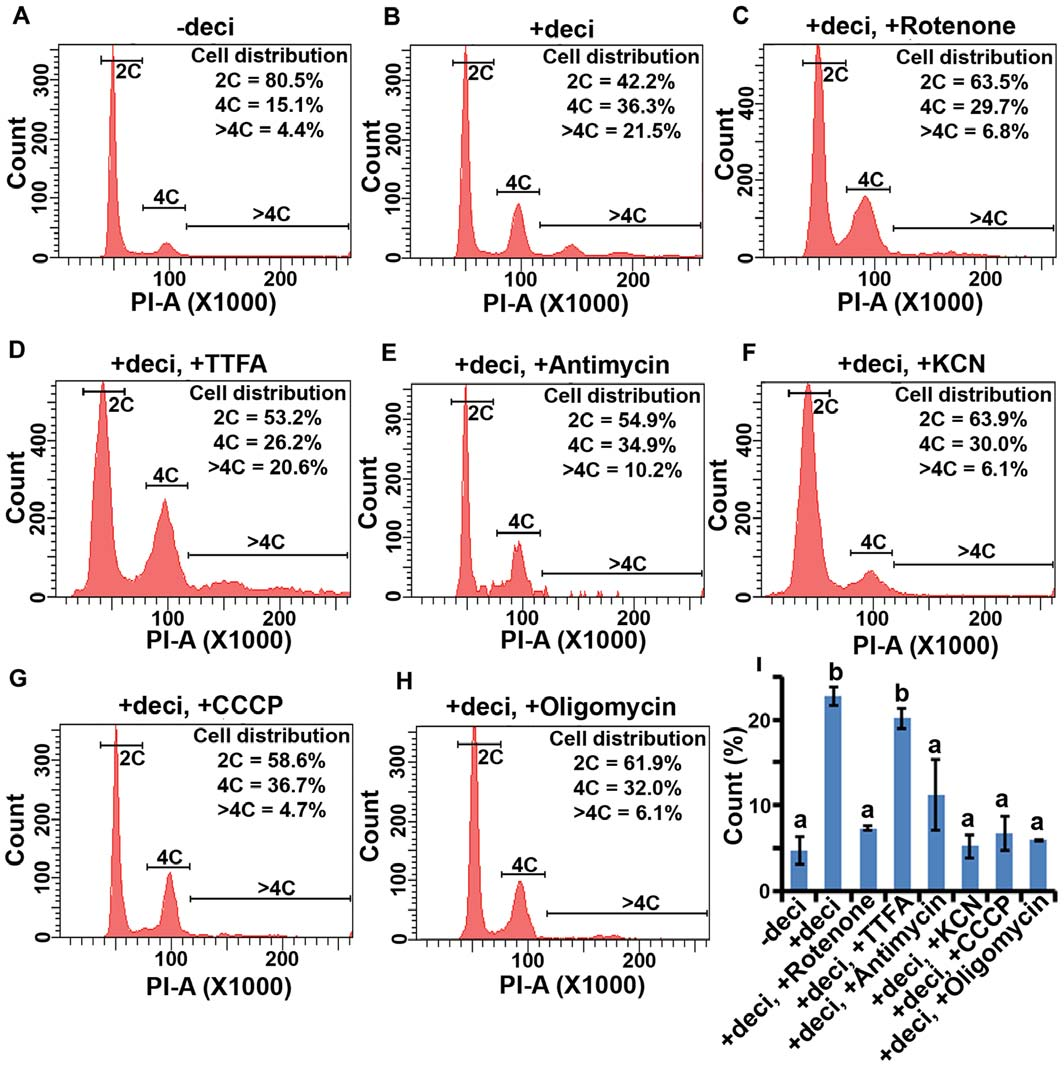
Effects of mitochondrial inhibitors on the development of polyploidy during *in vitro* decidualization. Day 4 uterine stromal cells in the culture were subjected to without (A) or with (B–H) stimulation for decidualization, as described in Materials and Methods. For the mitochondrial inhibitors study, at the time of decidual stimulation, cells were also treated without (B) or with different inhibitors: rotenone (0.2 µM) (C), TTFA (80 nM) (D), antimycin (2 µM) (E), KCN (500 µM) (F), CCCP (10 µM) (G) or oligomycin (12.5 nM) (H). Cells were collected after 5 days of decidualization and subjected to flow cytometric analysis for DNA quantitation. Quantitative analyses of cell distribution (%) based on the DNA content are shown as insets for each representative group. (I) Quantitative analysis of polyploid cell count (%) in presence or absence of mitochondrial inhibitors during decidualization. Results are expressed as mean ± SEM and representative of at least five independent experiments. The error bars represent standard errors. Values are statistically different (*P*<0.05, ANOVA followed by Newman-Keul's multiple range test) between a vs. b.

We further wanted to examine whether mitochondria-mediated functional activities play roles in the development of polyploidy during stromal cell decidualization *in vitro*. We applied several inhibitors for the respiratory chain [rotenone (complex I), TTFA (complex II), antimycin (complex III) and KCN (complex IV)], oxidative phosphorylation (oligomycin), and for uncoupling the respiratory chain and phosphorylation system (CCCP). Based on initial studies, selected doses of the inhibitors which show no obvious effects on cell viability were added in the culture during stromal cell decidualization, as described in Materials and Methods. Consistent to our previous report [29], there was a significant level of induction for decidual cell polyploidy, following the onset of decidualization when compared to the control (without decidualization) (Fig. 7A, B, I). However, the application of all of the above inhibitors, with the exception for TTFA, was able to show dramatic inhibition of polyploidy development during decidualization (Fig. 7C-I). These results further suggest that mitochondrial respiratory chain complex II is appeared to be not rate limiting in the above event. Because bi-nucleation is also associated with polyploidy development during decidualization, we also quantitatively analyzed binuclear cell development. Our analysis revealed a similar above results (data not shown). Overall, these results suggest that pharmacological inhibition of selective mitochondrial activity results in cessation of decidual cell polyploidy development.

**Figure 8:**
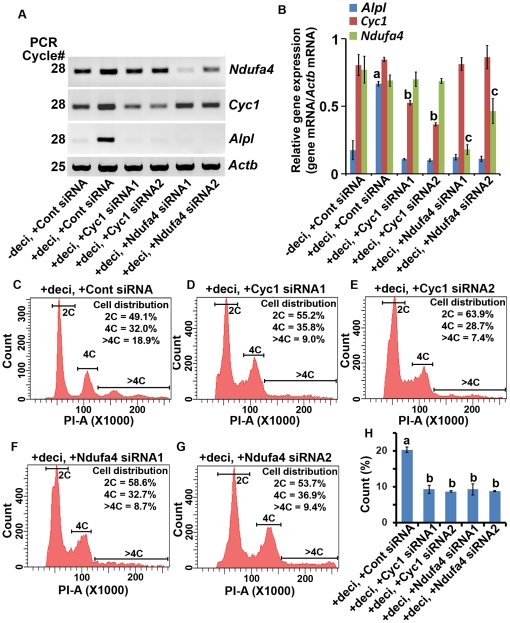
Effects of mitochondrial gene-specific inhibition on the development of polyploidy during *in vitro* decidualization. Day 4 uterine stromal cells in the culture were transfected with the control siRNA prior to without or with decidualization. In addition, two independent siRNAs for each of *Ndufa4* or *Cyc1* genes were also transfected in separate cultures prior to decidualization. Cells were collected after 5 days of decidualization. (A) RT-PCR analysis of expression for *Ndufa4*, *Cyc1* and *Alpl* (*alkaline phosphatase*). (B) Quantitative analyses of gene expression. The band intensities, as shown in the representative (A), were measured by densitometric analyses, and relative levels of mRNAs for gene-specific expression were determined after correction with *Actb*. These experiments were repeated at least three times with similar results. (C–G) Flow cytometric analysis. Quantitative analyses of cell distribution (%) based on the DNA content are shown as insets for each representative group. (H) Quantitative analysis of polyploid cell count (%) without or with inhibition of mitochondrial genes during decidualization. Results are expressed as mean ± SEM and representative of at least five independent experiments. The error bars represent standard errors. Values are statistically different (*P*<0.05, ANOVA followed by Newman-Keul's multiple range test) between a vs. b.

Furthermore, we also wanted to examine whether mitochondrial gene-specific perturbation affects polyploidy development during decidualization. We selected two gene-specific targets for the mitochondrial respiratory chain complex I (*Ndufa4*) and complex III (*Cyc1*). Suppression of these genes by two-independent siRNAs, we were able to demonstrate a significant and selective inhibition for each of the targets by at least 40–70% level of reduction (Fig. 8A, B). Interestingly, this inhibition for both the genes was consistent with the suppression of expression for the alkaline phosphatase (*Alpl*), a marker of stromal cell decidualization (Fig. 8A, B). The additional marker for the development of decidual cell polyploidy (cyclin D3) [29] was also similarly revealed like that of alkaline phosphatase (data not shown). Furthermore, the analysis of polyploidy development revealed that the above inhibition was able to cause significant abrogation of decidual cell polyploidy development (Fig. 8C-H). In addition, the analysis of binucleation also revealed a similar above results (data not shown). Overall, these results suggest that mitochondrial activity is critical for the development of decidual cell polyploidy. Moreover, mitochondrial-related genes (at least by Ndufa4 and Cyc1) are appeared to be positively correlated with the polyploidy-related gene at least by cyclin D3.

## Discussion

Uterine stromal cell decidualization begins with extensive stromal cell proliferation at the site of embryo implantation followed by its regional differentiation into specialized types of cells (decidual cells) with the acquisition of polyploidy. Recently, studies have shown that development of decidual cell polyploidy crucially directs appropriate control of uterine decidualization and early embryo implantation in mice [15], [44]. Utilizing the pure polyploid decidual cells as a model, we provide evidence to suggest that the “cell power house” mitochondria play a major role in decidual polyploidy development at the site of implantation, a novel mechanism which may be a common underlying cause of polyploidy development in other normal tissue biology. In addition, development of decidualization has been considered to be a pseudo-malignant growth and resembles various aspects of cancer, including its rapid development and growth, regulatory signaling, and the presence of polyploidy [4], [21], [45]. Despite these similarities, our studies suggest that decidual polyploid cells are highly enriched with mitochondrial activity, as opposed to the cancer cells, since according to Warburg's hypothesis, it has been defined that cancer cells lack mitochondrial activity for their survival [46].

### *In vivo* isolated polyploid decidual cells exhibit dramatic alteration of gene expression

The BSA density-gradient cell sedimentation technique has been successfully applied to isolate various mammalian cells [36], [47], [48]. The application of this technique also allowed us to obtain highly pure polyploid and non-polyploid decidual cell populations from the decidual bed. Subsequent use of these two cell populations for gene expression profiling revealed that they do possess distinct molecular signatures, suggesting that they are indeed not only morphologically, but also molecularly different. Gene expression profiling between the decidual polyploid and non-polyploid populations revealed a large list of genes (a total of 2222 genes) that undergo differential expression, suggesting a dramatic modulation of gene expression is needed during the transition from the non-polyploid to polyploid state, with a distinct morphological and functional shift within the decidual bed. In contrast, a recent microarray study demonstrated alterations of only a few genes in the hepatocytes with the change in ploidy levels [49], although both the hepatocytes and decidual polyploid cells exist with bi-nucleation. Uterine decidual polyploid cells are known to be short-lived and are typically terminally differentiated [11], while the polyploid hepatocytes are considered to be stable and less differentiated [1]. Thus, the alteration of a large number of genes in polyploid decidual may account for their short half-life.

### Numerous cell cycle regulatory molecules are specifically altered with decidual cell polyploidy

It has been well documented that cell cycle activity is an intrinsic part of the control of stromal cell decidualization [4], [11], [21], [29], [30]. Consistent with this suggestion, our study revealed a large list of altered genes (105 up-regulated genes and 11 down-regulated genes) that are related to the cell cycle in polyploid decidual cells (Fig. 4, Tables S3 and S4). Furthermore, it has been shown that the prerequisite transition from the mitotic cell cycle to the endocycle for decidual polyploidy development depends on the arrest prior to mitosis [11]. Interestingly, the present study revealed that polyploid decidual cells exhibit alterations of a significant number of genes (66 up-regulated genes and 6 down-regulated genes) that are specifically involved in the mitotic phase (Fig. 4, Tables S3 and S4). Furthermore, it is worth mentioning that *p57* (Fig. 2) and *Chk1* (Table S3) were up-regulated in polyploid decidual cells. *P57*, a growth arrest gene inhibits cdk1, has been shown to be associated with polyploidy development in trophoblast giant cells [50]. Furthermore, it has been shown that development of polyploidy involves DNA damage signals, primarily to trigger cell cycle arrest in the mitotic phase, through activation of the ATM/ATR-Chk1/Chk2 signal [51]. It is also interesting to note that *Ddb1* (damage specific DNA binding protein 1) is specifically expressed at the site of implantation, consistent with polyploidy distribution in the decidual bed (Fig. 3). In addition, our study revealed upregulation of 51 genes in polyploid cells, which are related to nuclear division (Table S2). Among the list of genes, several proteins such as Mad2L1, Mad2L2, Bub1, Bub1B, AurkA, and AurkB are known to play key roles in the spindle assembly checkpoint and maintenance of chromosomal stability [52]. Our analysis also revealed a list of genes that are specifically related to bi-nuclear polyploid cells (Table 1). In this regard, Tdo2 and Nsbp1 are detected in the decidual bed with bi-nuclear polyploid cells (Fig. 3). Mini-chromosome maintenance protein complex (Mcm2–7) functions as eukaryotic helicase in DNA replication [53]. Because polyploid cells involve continuous DNA synthesis, it is expected that the DNA replication complex may be repeatedly used. Consistently, several Mcm proteins (Mcm2, Mcm3, Mcm4, Mcm5, and Mcm7) are up-regulated in polyploid decidual cells (Table S3). FoxM1 transcription factor plays a critical role in cell proliferation, differentiation, and transformation [54]. Although, FoxM1 was not directly altered in polyploid cells, FoxM1 signaling networks, consisting of 14 genes (see Table S3), were specifically up-regulated with polyploidy, suggesting a potential of this signaling in relation to polyploidy.

### Decidual polyploid cells exhibit lack of apoptosis and immune suppression properties

The decidua is thought to provide immune protection to the embryo from immunological responses of the mother and to regulate trophoblast invasion. Previously, it has been reported that immune responsive genes were suppressed in the uterine decidual bed at the site of the implanting embryo, suggesting the process of implantation is immunologically privileged during early pregnancy, and immune suppression may be necessary in regulating the uterine environment during the progression of embryo implantation [55]. Consistent with this finding, our study also observed that a large number of immune-regulated genes (254 genes) are specifically down-regulated in polyploid decidual cells as compared to non-polyploid cells. Previously, it has been shown that uterine decidual polyploid cells specifically lack apoptosis [56]. In this regard, we found that genes related to apoptosis (135 genes) are also specifically suppressed in polyploid decidual cells as compared to non-polyploid cells (Fig. 4).

### Increased mitochondrial activity determines the development of decidual cell polyploidy

Mitochondria are multitasking organelles involved in ATP synthesis, reactive oxygen species (ROS) production, calcium signaling, and apoptosis. Mitochondrial dysfunction has been shown to be linked with various physiological abnormalities, in particular with ageing and male infertility [57], [58]. However, the mitochondrial role in female reproduction, specifically with uterine decidualization, remains largely unknown. Here, we suggest that hyper-activation of mitochondria is positively linked with polyploidy development. Consistently, a large number of mitochondrial genes exhibited upregulation in polyploid decidual cells (Fig. 4, Table S3). Among these, several candidate genes were indeed specifically verified in polyploid decidual cells (Fig. 5A,B). Additionally, several mitochondrial genes appeared to be either not changed [Nd1, Nd2, Nd3, Nd4l, Cytb, Atp6, Cox1 and 2 (cytochrome C oxidase subunit 1 and 2)] or downregulated (Cmpk2 and Ucp2) in polyploid cells, suggesting that polyploidy-related increase in gene expression are not simply due to the increase in mitochondrial mass (Fig. 6A,B). Moreover, several mitochondrial genes (Eln, Tmtc1, Ak1, and Me1) revealed that they are specifically up-regulated cell-specifically at the site of implantation, consistent with polyploidy in the decidual bed (Fig. 5C). Furthermore, it should be noted that the glycolysis pathway was probably not utilized in the polyploid cells, since glycolysis-promoting enzyme *Pfkfb3* was specifically down-regulated by 4 fold in polyploid cells (Table S1). This enzyme generates fructose-2,6-bisphosphate, the most potent activator of Pfk1, a master regulator of glycolysis [59]. The development of polyploidy has been reported to occur during the increase in oxidative stress and reactive oxygen species (ROS) production [60], [61], [62], [63]. Nox4, an enzyme necessary for ROS production, has also been shown to be associated with polyploidy development [64]. Our analysis revealed that *Nox4* is indeed up-regulated in polyploid cells and expressed in the decidual bed consistent with polyploidy (Figs. 2 and 3). Most interestingly, pharmacological (Fig. 7) and genetic (Fig. 8) perturbation of mitochondrial activity caused dramatic attenuation of polyploidization, suggesting that mitochondrial events are intimately associated with polyploidy.

### Regional expression of polyploidy-related genes appeared to be developmentally controlled

Previously, it was shown that regional uterine decidualization is predominantly developmentally controlled. For example, Hoxa-10, a developmentally regulated homeobox transcription factor, is highly expressed in decidualizing stromal cells, and targeted deletion of *Hoxa-10* in mice shows severe decidualization defects with aberration of regional expression of different decidualization marker genes [19]. In the present study, we report a differential expression with regional distribution for several polyploidy related genes in the decidual bed at the site of implantation. This may suggest that many of the polyploidy-related genes could be developmentally controlled. In this regard, it is interesting to mention that polyploidy related gene (Nsbp1) is aberrantly expressed in the decidual bed with defective decidualization in *Hoxa-10* null mice (Ma X and Das SK, unpublished observation). Collectively, a host of polyploidy-related regionally-expressed genes may be necessary to induce multiple molecular/signaling pathways for propagating a normal decidual development during the early post-implantation period.

### Polyploid-related genes may determine the female fertility

The identification of global gene expression profiles specifically for decidual polyploid cells should provide novel insight into the cellular and molecular mechanisms underlying developmental aspects of polyploidy during stromal cell decidualization. Here we provide further evidence to suggest that mitochondrial activity is essential to the development of decidual cell polyploidy. Because the development of decidual cell polyploidy appears to be crucial for successful implantation, our study may also suggest that any mitochondrial dysfunction such as with aging could result in defective decidual cell polyploidy in early pregnancy leading to infertility.

## Supporting Information

Figure S1 **Analysis of cyclin D3 expression in the isolated polyploid and non-polyploid cell populations.** Cells were subjected to cytospin on the slides and then analyzed by immunostaining with cyclin D3. Cells incubated with non-immune serum (IgG) are shown as control. Dark-brown stain indicates the expression of cyclin D3. Pictures are shown at 400X.
(TIF)

Figure S2 **Gene clustering.** Data obtained from microarray analysis for differential gene expression between the polyploid (P1, P2, and P3) and non-polyploid (N1, N2, and N3) decidual cell populations was used to generate a cluster analysis. Each *vertical line* represents a single gene. Upregulation and downregulation in expressions are represented as different levels of shading (red, yellow, and blue) in the heatmap. The degree of color saturation reflects the magnitude of gene expression, as indicated by color scale.
(TIF)

Figure S3 ***In situ*** **hybridization analysis of expression for the** ***Ptgs2*** **gene at the sites of embryo implantation on days 5 (D5) and 7 (D7) of pregnancy.** Dark-field photomicrographs of representative uterine cross-sections hybridized with antisense probes are shown. M, mesometrial pole; AM, anti-mesometrial pole; e, embryo. Pictures are shown at 40X.
(TIF)

Figure S4 **Immunohistochemical analysis of cyclin D3 on day 8 embryo implantation site.** M, mesometrial pole; AM, anti-mesometrial pole; e, embryo; sdz, secondary decidual zone. The insets shown in the mesometrial-antimesometrial barrier region or in the antimesometrial region in panel a (at 40X) are presented in the respective right panels: b or c (at 400X).
(TIF)

Figure S5 **Functional networks of mitochondrial genes that are specifically up-regulated in polyploid decidual cells.** Ingenuity Pathway Analysis (IPA) was performed to obtain mitochondrial gene networks and an overlapping relationship between the networks, based on 128 mitochondrial genes that are induced in polyploid cells (Table S3). Genes that are marked in red actually represent induced genes for polyploid populations, while increasing intensities of red indicate higher orders of expression. Each network is displayed graphically as nodes (gene or gene product) and edges (the biological relationships between nodes, including the functional or physical interactions). The overlapping network is generated based on each constructed network that is bound by commonly appearing genes. The shape of the objects represents whether the protein is a cytokine, growth factor, chemical/drug/toxicant, enzyme, etc. as indicated in the figure.
(TIF)

Figure S6 **Analysis of mitochondria in relation to decidual cell polyploidy developed** ***in vitro*** **.** A. Mitochondrial mass analysis. Confocal microscopic analysis was performed on cells after staining with Mitotracker *Red*, as described in Materials and Methods. B. Quantitation of mitochondrial mass. The area of mitochondrial staining per cell was determined using the Image J program available at http://imagej.nih.gov/ij (NIH, USA). Results are expressed as fold change (mean ± SEM), as compared to non-polyploid cells (control). Data were analyzed after counting of at least 30 to 40 cells in each group from three independent experiments.
(TIF)

Table S1 Complete list of up- or down-regulated genes in polyploid (P) cells as compared to non-polyploid (N) cells.
(XLS)

Table S2 Primers used for RT-PCR analyses.
(DOC)

Table S3 Enrichment analysis of polyploidy-related up-regulated genes generated by TOPPFUN program.
(XLS)

Table S4 Enrichment analysis of polyploidy-related down-regulated genes generated by TOPPFUN program.
(XLS)
