# Supplementary figures and images for: Decidual Cell Polyploidization Necessitates Mitochondrial Activity
Source: PLoS One. 2011 Oct 25;6(10):e26774. doi: 10.1371/journal.pone.0026774 (PMC3201964; doi:10.1371/journal.pone.0026774)

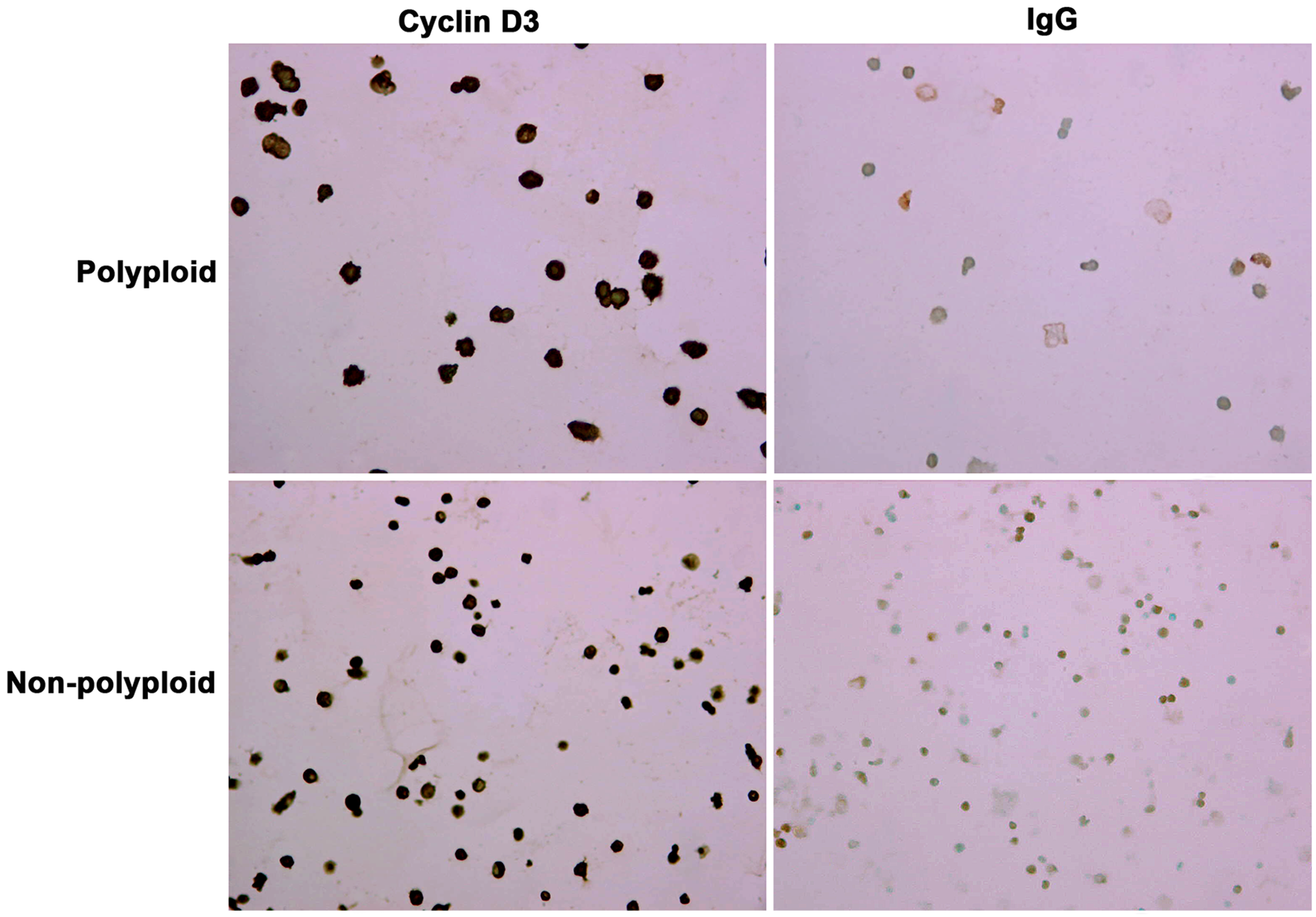

Supplement: Figure S1 — Analysis of cyclin D3 expression in the isolated polyploid and non-polyploid cell populations. Cells were subjected to cytospin on the slides and then analyzed by immunostaining with cyclin D3. Cells incubated with non-immune serum (IgG) are shown as control. Dark-brown stain indicates the expression of cyclin D3. Pictures are shown at 400X. (TIF) [file pone.0026774.s001.tif]

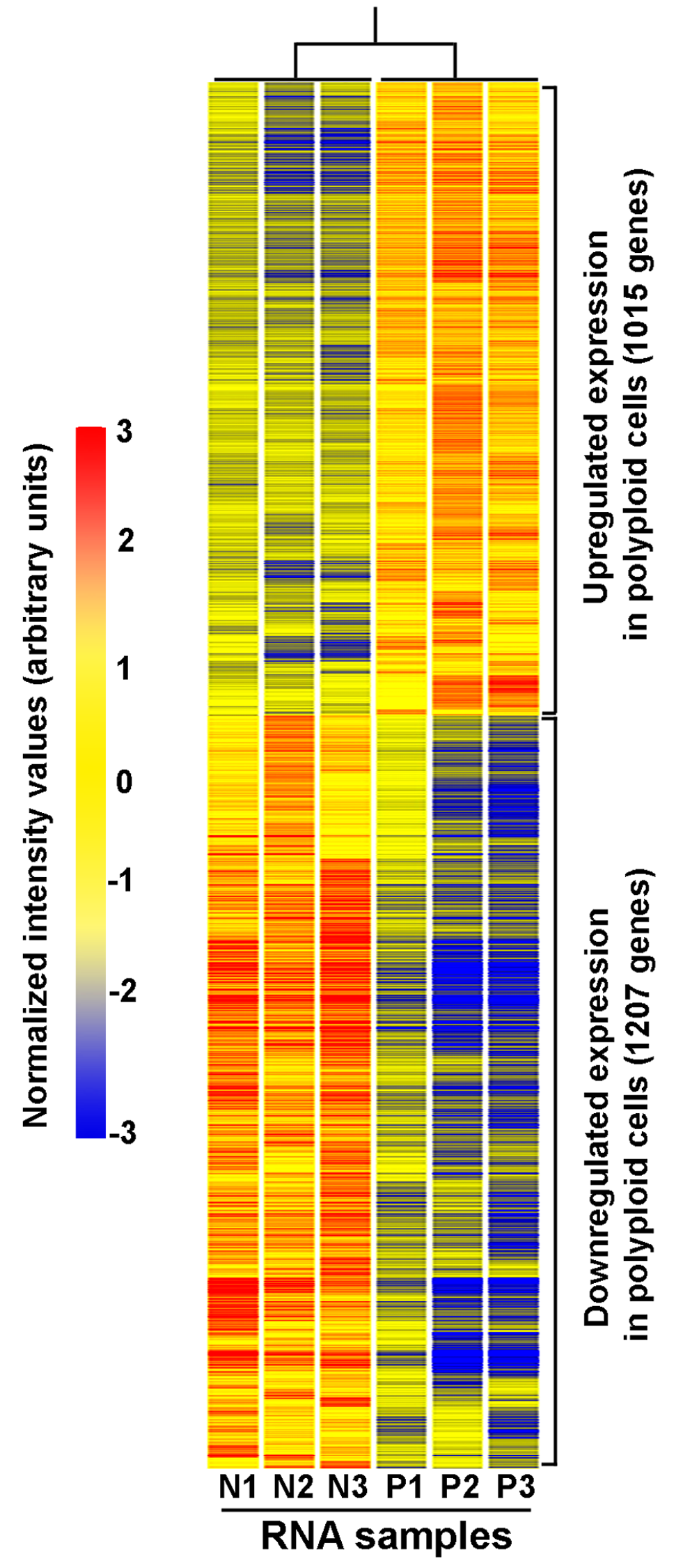

Supplement: Figure S2 — Gene clustering. Data obtained from microarray analysis for differential gene expression between the polyploid (P1, P2, and P3) and non-polyploid (N1, N2, and N3) decidual cell populations was used to generate a cluster analysis. Each vertical line represents a single gene. Upregulation and downregulation in expressions are represented as different levels of shading (red, yellow, and blue) in the heatmap. The degree of color saturation reflects the magnitude of gene expression, as indicated by color scale. (TIF) [file pone.0026774.s002.tif]

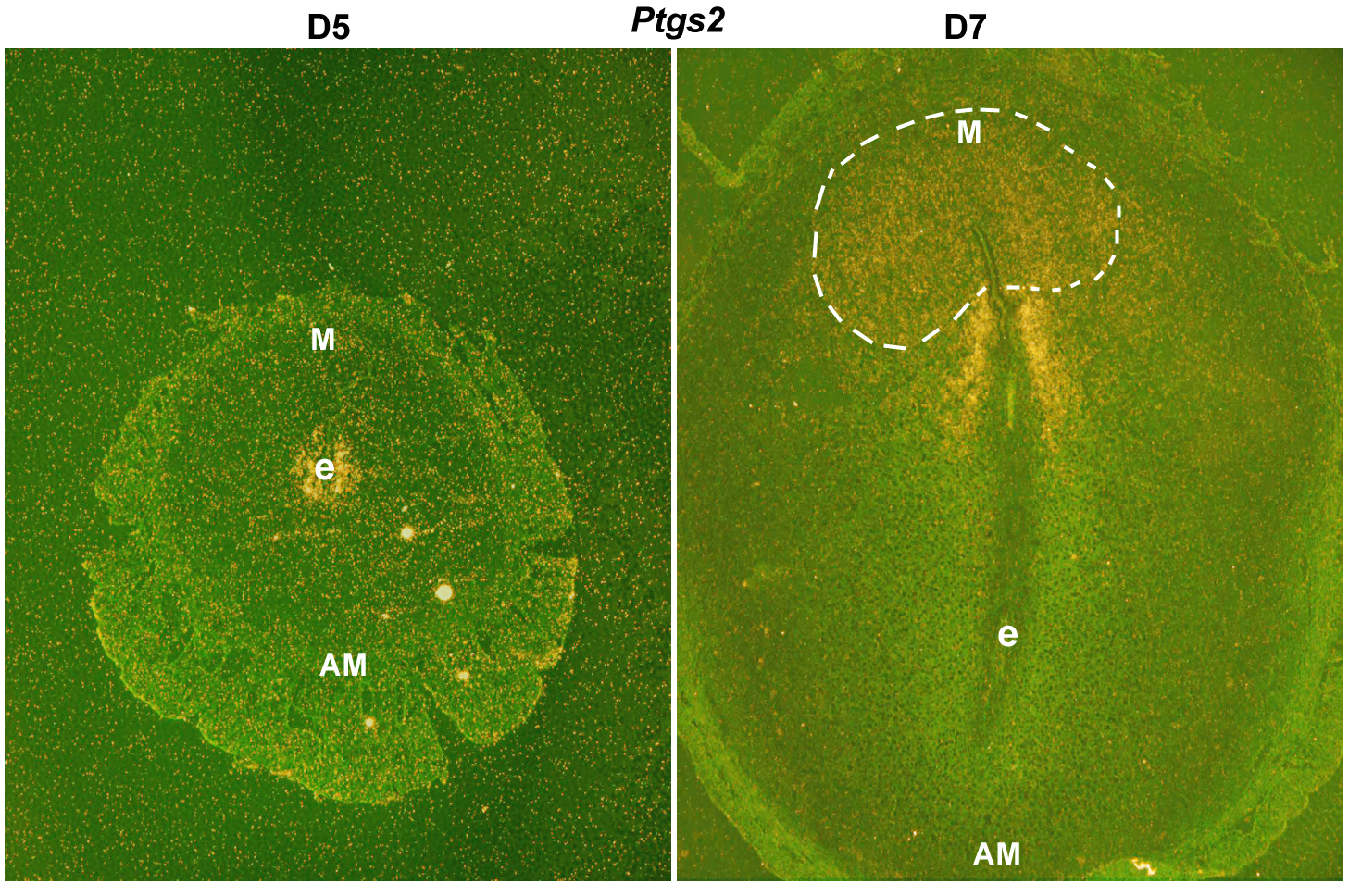

Supplement: Figure S3 — In situ hybridization analysis of expression for the Ptgs2 gene at the sites of embryo implantation on days 5 (D5) and 7 (D7) of pregnancy. Dark-field photomicrographs of representative uterine cross-sections hybridized with antisense probes are shown. M, mesometrial pole; AM, anti-mesometrial pole; e, embryo. Pictures are shown at 40X. (TIF) [file pone.0026774.s003.tif]

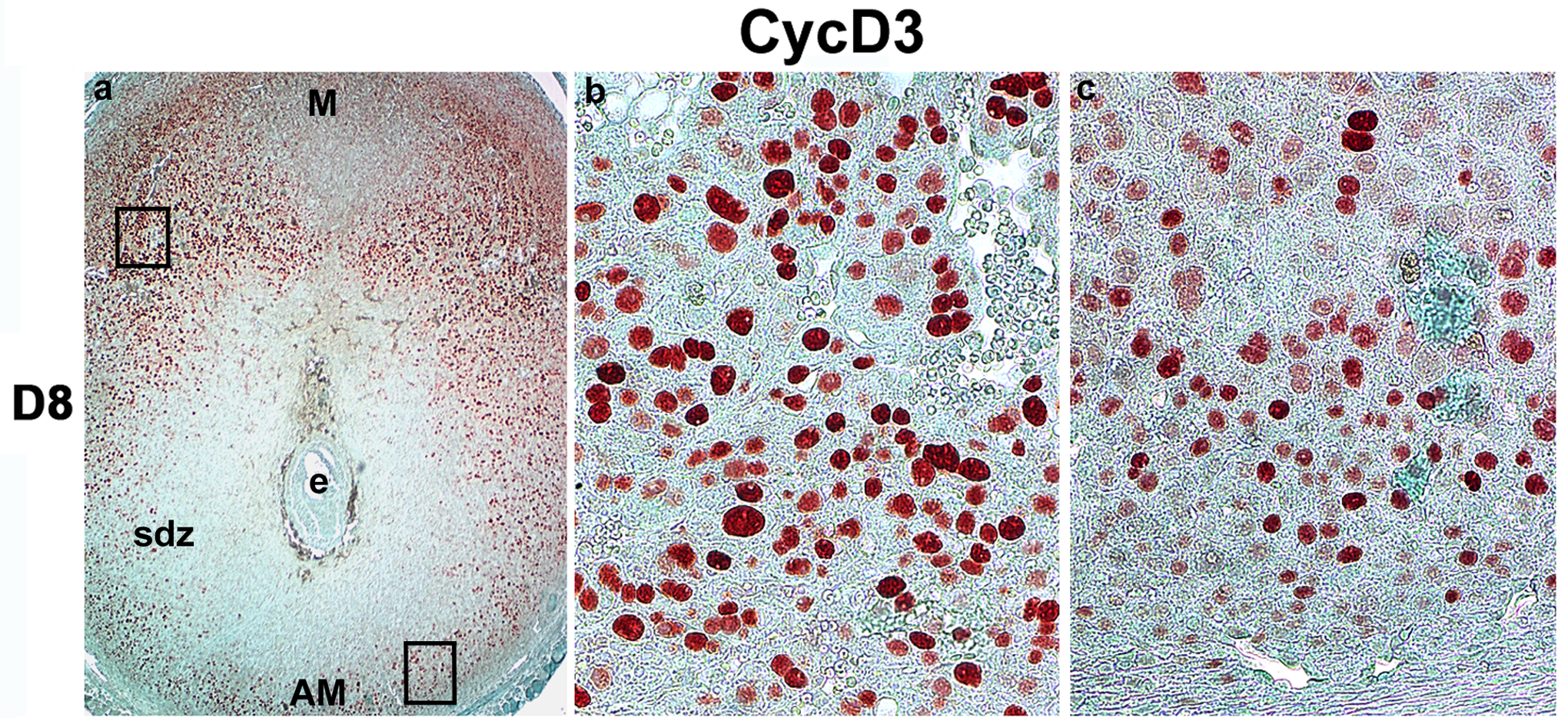

Supplement: Figure S4 — Immunohistochemical analysis of cyclin D3 on day 8 embryo implantation site. M, mesometrial pole; AM, anti-mesometrial pole; e, embryo; sdz, secondary decidual zone. The insets shown in the mesometrial-antimesometrial barrier region or in the antimesometrial region in panel a (at 40X) are presented in the respective right panels: b or c (at 400X). (TIF) [file pone.0026774.s004.tif]

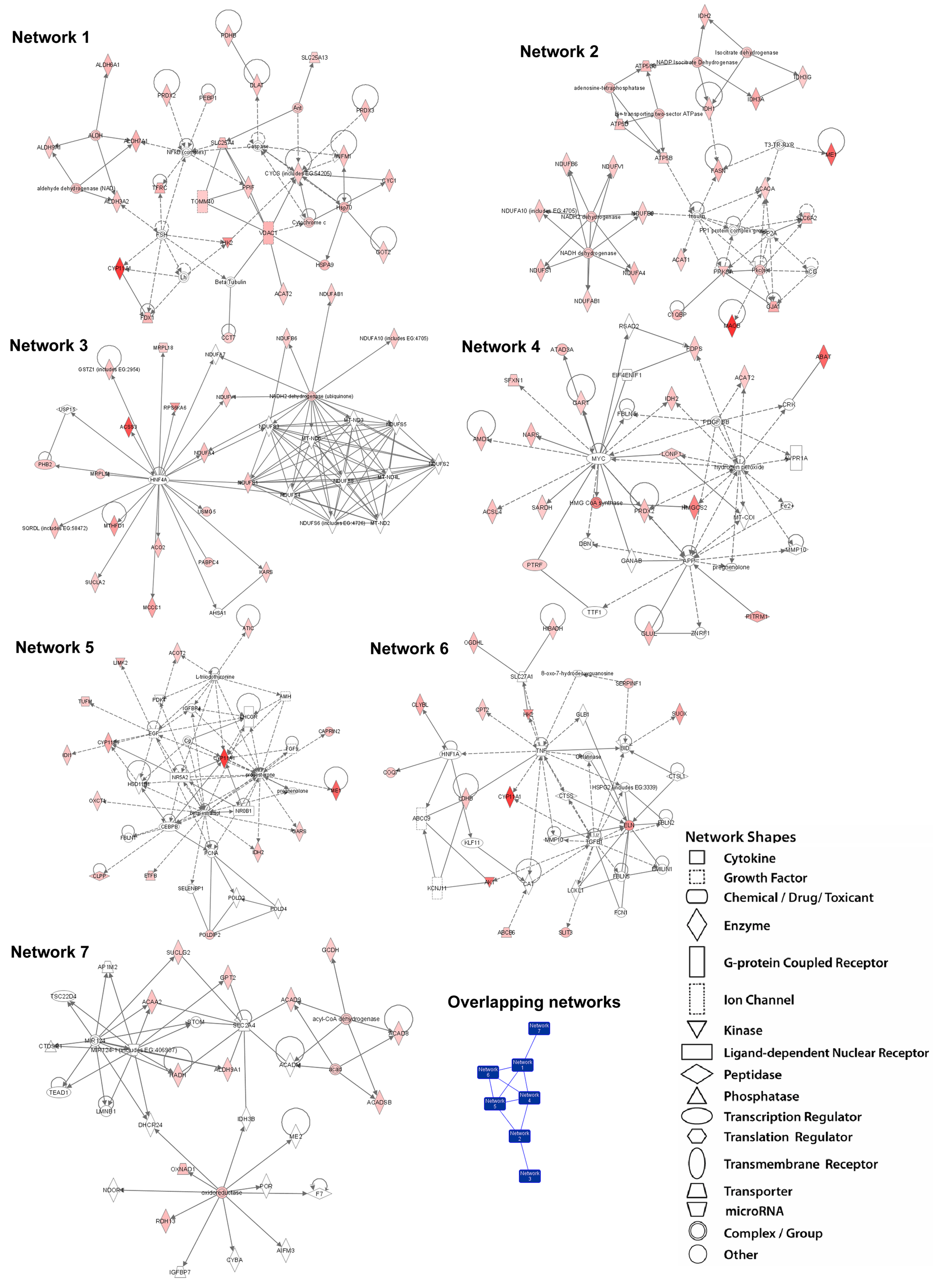

Supplement: Figure S5 — Functional networks of mitochondrial genes that are specifically up-regulated in polyploid decidual cells. Ingenuity Pathway Analysis (IPA) was performed to obtain mitochondrial gene networks and an overlapping relationship between the networks, based on 128 mitochondrial genes that are induced in polyploid cells (Table S3). Genes that are marked in red actually represent induced genes for polyploid populations, while increasing intensities of red indicate higher orders of expression. Each network is displayed graphically as nodes (gene or gene product) and edges (the biological relationships between nodes, including the functional or physical interactions). The overlapping network is generated based on each constructed network that is bound by commonly appearing genes. The shape of the objects represents whether the protein is a cytokine, growth factor, chemical/drug/toxicant, enzyme, etc. as indicated in the figure. (TIF) [file pone.0026774.s005.tif]

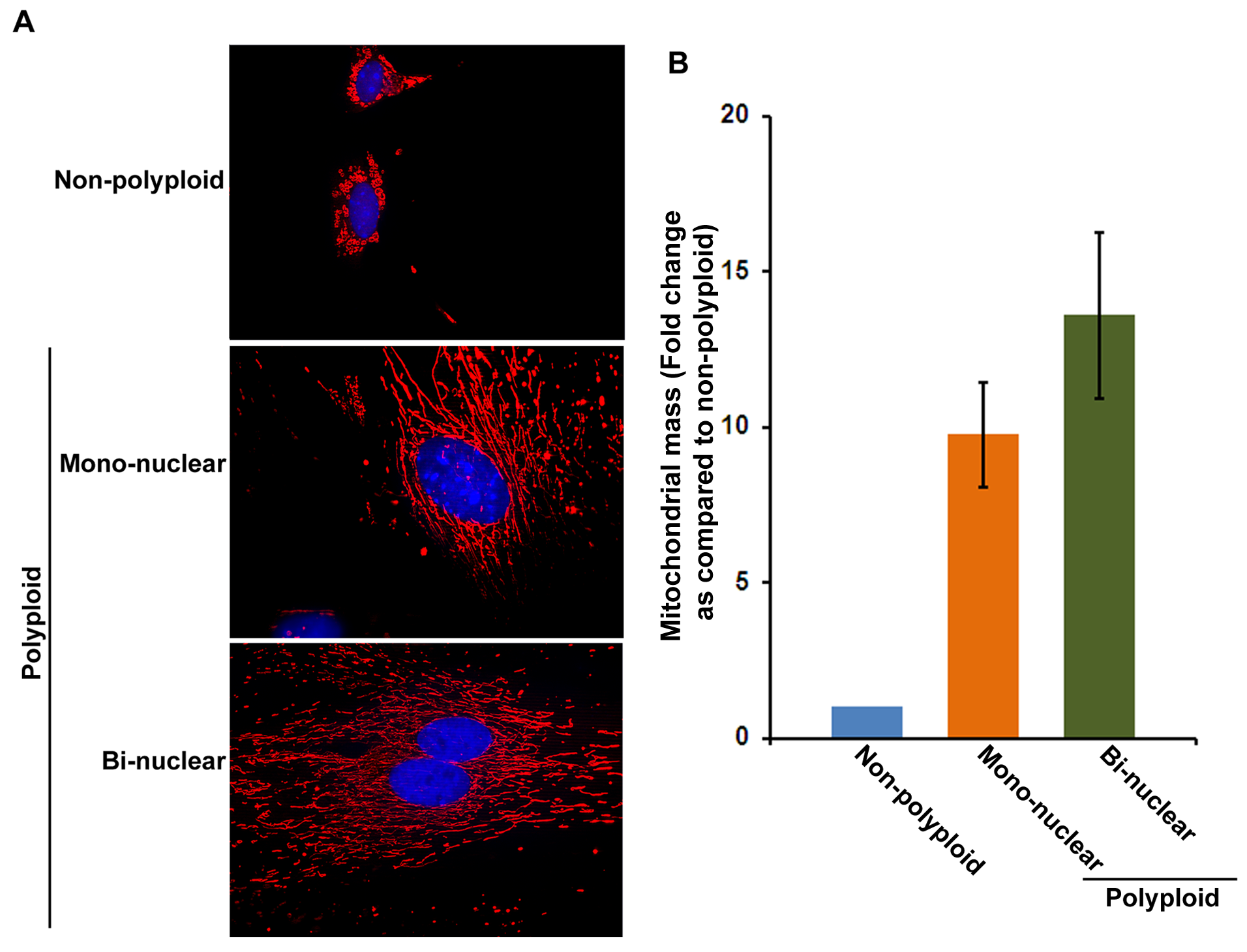

Supplement: Figure S6 — Analysis of mitochondria in relation to decidual cell polyploidy developed in vitro . A. Mitochondrial mass analysis. Confocal microscopic analysis was performed on cells after staining with Mitotracker Red, as described in Materials and Methods. B. Quantitation of mitochondrial mass. The area of mitochondrial staining per cell was determined using the Image J program available at http://imagej.nih.gov/ij (NIH, USA). Results are expressed as fold change (mean ± SEM), as compared to non-polyploid cells (control). Data were analyzed after counting of at least 30 to 40 cells in each group from three independent experiments. (TIF) [file pone.0026774.s006.tif]
