# Supplementary material for: Decidual Cell Polyploidization Necessitates Mitochondrial Activity
Source: PLoS One. 2011 Oct 25;6(10):e26774. doi: 10.1371/journal.pone.0026774 (PMC3201964; doi:10.1371/journal.pone.0026774)
Supplement: Table S2 — Primers used for RT-PCR analyses. (DOC) [file pone.0026774.s008.doc]

**Table S2**: Primers used for RT-PCR analyses

| Name of genes | Sequence |
| --- | --- |
| *Tdo2* | Tdo2-L: CATGGCTGGAAAGAACACCT  Tdo2-R: CCTTGTACCTGTCGCTCACA |
| *Prc1* | Prc1-L: AGCCTGTGGAGGCAATTATG  Prc1-R: TCCGTGCTTTCAACTCCTCT |
| *Acad9* | Acad9-L: GGATGCTAGACCAACCAGGA  Acad9-R: ACAGTCCGGCCAAAGTAATG |
| *Aurkb* | Aurkb-L: GCCAGAAGTTGGCTGAGAAC  Aurkb-R: CTCCCTGCAGACCTAACAGC |
| *Anln* | Anln-L: GCCTGCACTCACTTCTTTCC  Anln-R: CTGAGTTCAGCCCTCTGTCC |
| *Neo1* | Neo1-L: CTCTACCGCTGCATTGTTGA  Neo1-R: TCCATGGATTCGTGAGCATA |
| *Nsbp1* | Nsbp1-L: GATGGGAAATGCAAAGAGGA  Nsbp1-R: TCTTCTGCCTCCACCTTGTT |
| *Me1* | Me1-L: GGAACCCACCTCTGAGACAA  Me1-R: GAAAGCTTCTGCACCCTCAC |
| *Tmtc1* | Tmtc1-L: CAAGCTTGGCTTCCCTACTG  Tmtc1-R: GCTAAAACCTGTGCCAGAGC |
| *Ak1* | Ak1-L: GAGACCATGACCCAACGACT  Ak1-R: AGCCAGAGGGAGACAAGACA |
| *Nox4* | Nox4-L: GCATCTGCATCTGTCCTGAA  Nox4-R: ACCACCTGAAACATGCAACA |
| *Eln* | Eln-L: TCAGATGGCTCCTCACACTG  Eln-R: AACCCAAAGAGCACACCAAC |
| *Acss3* | Acss3-L: TACAAGCCCTGGACCAAAAC  Acss3-R: ACTCCACCTTCCTTCCTGGT |
| *Tfrc* | Tfrc-L: CAGCATTGGTCAAAACATGG  Tfrc-R: TTTTCTGCAGCAGCTCTTGA |
| *Abat* | Abat-L: TGGTACCGGAGTAAGGAACG  Abat-R: CTTGCCTGTACAGCCTCCTC |
| *Limk2* | Limk2-L: TCAAAGCTGGAGGACTCGTT  Limk2-R: CCTCCCAGAATCTCAATCCA |
| *Ddb1* | Ddb1-L: AGCAACGAACAAGGCTCCTA  Ddb1-R: GCTTTGGGCTCTTGAGACAC |
| *P57* | P57-L: CTGGGACCTTTCGTTCATGT  P57-R: GTGGGGGCTTTTACTCAACA |
| *Serpinb6b* | Serpinb6b-L: TGGATAAATGCAGTGGCAAA  Serpinb6b-R: TTGCACAGGTTTCACCACAT |
| *Pdgfra* | Pdgfra-L: ACCTCCCACCAGGTCTTTCT  Pdgfra-R: CTCCCGTTATTGTGCAAGGT |
| *Pdgfc* | Pdgfc-L: GTGCCAGGAAAGCAGACTTC  Pdgfc -R: ACAGGCACAATTTCCTCCAC |
| *Fmo2* | Fmo2-L: CTCACCTGCCACTCAAGTCA  Fmo2-R: GTCTCCGTGAGCTCCTTCAC |
| *Dcn* | Dcn-L: GCTTCCTACTCGGCTGTGAG  Dcn-R: GGCGGCATTTGACTTTATGT |
| *Aldh1a2* | Aldh1a2-L: GGCTGGGCTGATAAAATTCA  Aldh1a2-R: GTCCAAGTCAGCATCTGCAA |
| *Ccr2* | Ccr2-L: CCTGCAAAGACCAGAAGAGG  Ccr2-R: GTGAGCAGGAAGAGCAGGTC |
| *Chi3l3* | Chi3l3-L: TGAAGGAGCCACTGAGGTCT  Chi3l3-R: CCAGCTGGTACAGCAGACAA |
| *Ms4a4c* | Ms4a4c-L: GAGGTGCTCCACCTTCAGAG  Ms4a4c-R: TCCAAACCCTTGGTGATTGT |
| *Il1b* | Il1b-L: GAGTGTGGATCCCAAGCAAT  Il1b-R: CTGCCTAATGTCCCCTTGAA |
| *Clec4e* | Clec4e-L TGCTACAGTGAGGCATCAGG  Clec4e-R: CATGCATGTAAGCCATGTCC |
| *Cybb* | Cybb-L: ATAGGCGTTTTCCTGTGTGG  Cybb-R: ­­­AAAGGTGACCACACCCAGAG |
| *Actb* | Actb-L: GTGGGCCGCTCTAGGCACCAA  Actb-R: CTCTTTGATGTCACGCACGATTTC |
